# Mitochondrial DNA of Sardinian and North-West Italian Populations Revealed a New Piece in the Mosaic of Phylogeography and Phylogeny of *Salariopsis fluviatilis* (Blenniidae)

**DOI:** 10.3390/ani12233403

**Published:** 2022-12-02

**Authors:** Ilenia Azzena, Fabio Scarpa, Chiara Locci, Piero Cossu, Alessio Niffoi, Flavio Orrù, Stefano Bovero, Giuseppe Sotgiu, Daria Sanna, Marco Casu

**Affiliations:** 1 Dipartimento di Scienze Biomediche, Università di Sassari, Viale San Pietro 43/B, 07100 Sassari, Italy; 2 Dipartimento di Medicina Veterinaria, Università di Sassari, Via Vienna 2, 07100 Sassari, Italy; 3 ENAS Ente Acqua della Sardegna, Servizio Qualità Acqua Erogata, 09123 Cagliari, Italy; 4 Independent researchers “Zirichiltaggi” Sardinia Wildlife Conservation NGO, 07100 Sassari, Italy

**Keywords:** freshwater blenny, mtDNA, control region, phylogeny, *Salariopsis fluviatilis*

## Abstract

**Simple Summary:**

The present study provides new molecular data on populations of the freshwater blenny *Salariopsis fluviatilis* from Italian geographic areas (north-western regions and Sardinia Island) so far never investigated and uses five species delimitation methods in order to shed further light on the Mediterranean phylogeography of this fish and give a more comprehensive scenario of its taxonomic status. Our findings clarified the phylogeographical patterns of the northern Italian populations of *Salariopsis fluviatilis sensu stricto*, considering the Po River basin and some Tyrrhenian basins of the Liguria region on the other side of the Ligurian Alps. The dispersal pathways of the island of Sardinia were also investigated suggesting that patterns of genetic structuring for this species are probably linked to Pleistocene glacial and interglacial periods. Results obtained supported previous studies in evidencing the occurrence of a species complex for *Salariopsis fluviatilis* with at least three taxonomic units: *Salariopsis fluviatilis sensu stricto*, *Salariopsis* cf. *fluviatilis* diffused in the Middle East and a further taxonomic entity from the Iberian Guadiana River basin whose tributaries flow in the Atlantic Ocean. For what concerns *Salariopsis fluviatilis sensu stricto*, two divergent groups of populations were reported in the present study, being the first diffused in western Mediterranean areas and the second in western Adriatic and eastern Mediterranean areas.

**Abstract:**

The genus *Salariopsis* (Blenniidae) comprises freshwater blenny fish that inhabits Mediterranean Sea, Black Sea, and north-east Atlantic areas. Three species were formally described to date: *Salariopsis fluviatilis*. *S. economidisi*, and *S. atlantica*. In this study, 103 individuals were collected from different Italian regions (Sardinia, Liguria, Piedmont, Lombardy) and analyzed using the mtDNA Control Region and the ribosomal 16s gene. We aimed (i) to depict the phylogeographic patterns of *S. fluviatilis* in northern Italy and Sardinia and (ii) to compare the genetic structure of Italian samples with those from other Mediterranean regions. Results obtained showed the presence of a well-supported genetic structuring among Italian *S. fluviatilis* populations, shedding new light on the phylogeographic patterns of northern Italian populations of *S. fluviatilis sensu stricto* across the Ligurian Alpine ridge and the Sardinia Island-mainland dispersal patterns. Furthermore, our species delimitation analysis was consistent in supporting results of previous research about the presence of genetic differentiation among *S. fluviatilis*, evidencing: (i) a large group of *S. fluviatilis sensu stricto* that includes two sub-groups (Occidental and Oriental), (ii) one group comprising populations from the Middle East of a taxonomic entity corresponding to *Salariopsis* cf. *fluviatilis*, and (iii) one group of Iberian individuals from the Guadiana River.

## 1. Introduction

The freshwater blenny, *Salariopsis fluviatilis* (Asso, 1801) (Actinopterygii: Blenniiformes: Blenniidae), is a small benthic fish that inhabits river basins and lakes around the Mediterranean Sea. It belongs to the recently described genus *Salariopsis* Vecchioni, Ching, Marrone, Arculeo, Hundt & Simons, 2022, proposed by Vecchioni and co-authors [1] in order to differentiate freshwater species previously referred to the genus *Salaria* Forsskål, 1775. Nowadays, the genus *Salariopsis* includes freshwater species whose distribution is across the eastern Atlantic coasts (Morocco), the Mediterranean Sea, and the Black Sea [2,3].

Worth noting, in the same year Duquenne-Delobel and co-authors [4] revalidated the genus *Ichthyocoris* Bonaparte, 1840 for the freshwater blennies. Although a debate is still ongoing [5], at the current state of the art we chose to use the genus *Salariopsis* as senior synomin, based on both the prior date of publication and the validation of the genus given by Vecchioni and co-authors [5].

*Salariopsis fluviatilis*—whose origin has been enrolled to a marine ancestor [6,7,8,9,10]—can show both morphological and behavioral differences in its populations, along with diversity in the adults/juveniles ratio, in response to the extended range of distribution and the different environmental factors, such as levels of predation, intraspecific competition, water flow, and lacustrine vs. river habitats [6,7,11,12,13,14]. For these reasons, different studies (see e.g., Belaiba et al. [15], Wagner et al. [16] and references therein) give rise to the suspicion that *S. fluviatilis* could be composed of an articulated species complex.

In this context, in addition to *S. fluviatilis*, two other freshwater blennies species have been formally described to date, characterized by a very limited distribution: *Salariopsis economidisi* (Kottelat, 2004), endemic to Lake Trichonis in Greece [2], and *Salariopsis atlantica* Doadrio, Perea & Yahyaoui, 2011, endemic to the Seboui river basin in Morocco [17].

Although *S. fluviatilis* shows, within the genus, the widest range of distribution, its populations are generally small and highly localized [18,19,20,21,22,23], suggesting that this species might have undergone high fragmentation phenomena that may have also produced relevant genetic divergences, at least in the most isolated populations, as likely happened to the other two freshwater *Salariopsis* species, which show confined (i.e., *S. atlantica*) or even point-like (i.e., *S. economidisi*) distributions.

Similar to other freshwater fish living in fragmented habitats, *S. fluviatilis* is highly susceptible to several anthropomorphic disturbances and to global environmental change effects. Nevertheless, even though it has been considered vulnerable or endangered in several countries and reported in Appendix III of the Bern Convention, *S. fluviatilis* has been evaluated by the International Union for Conservation of Nature (IUCN) as data deficient (IUCN 2013), due to a lack of data on its abundance and population trend.

Despite the potential interest of this species from phylogeographic, taxonomic and conservation perspectives, not many studies have been performed to depict the genetic structure and phylogeography of *S. fluviatilis* [7,8,9,15,16,20,24,25]. Most of these studies focused on the mitochondrial phylogenetic and phylogeographic patterns of the whole genus *Salariopsis* in the Mediterranean area, with inferences reported also for *S. fluviatilis.* Wagner et al. [16], in particular, supported the presence of at least six divergent genetic lineages within the *S. fluviatilis* populations: (1) Middle East (Israel, Syria and southern Turkey); (2) Guadiana (Guadiana basin with the Zùjar and Esteras rivers that flow on the European Atlantic coast); (3) Algeria-Verde (in southern Spain and Algeria with the Verde River and Oued Boughzazene); (4) Occidental basin (Algeria, Spain, mainland France, Corsica and Switzerland), (5) North Oriental basin (Italy, Croatia, Albania, Greece and western Turkey), and (6) the Island of Crete (Greece). The results of Wagner et al. [16] also support the results of other studies (see e.g., Belaiba et al. [15]), suggesting the presence of more than one taxonomic unit under the binomen *Salariopsis fluviatilis*. Remarkably, based on the results of two methods of species delimitation, Belaiba et al. [15] suggest that the populations of *S. fluviatilis* from the Middle East Mediterranean region should be regarded as a differentiated species, i.e., *Salaria* cf. *fluviatilis*. However, in light of the description of the new genus *Salariopsis* [1,5], we refer to it as *Salariopsis* cf. *fluviatilis* hereafter.

Furthermore, two main studies focusing specifically on the nuclear genetic variation of *S. fluviatilis* in the western Mediterranean [20,25] suggest that Corsica Island may have served as a glacial refuge during the last glacial maximum [25], as well as the presence of specific genetic lineages in the Iberian Peninsula [20]. In particular, Laporte et al. [25] argued that the genetic structuring retrieved between western and eastern populations of *S. fluviatilis* in the Mediterranean would be the product of the recent spreading of individuals from the two glacial refuges of the Iberian Peninsula and Corsica Island. However, in this study, the lack of samples from Liguria (i.e., north-west Italy) probably affected the possibility to fully reconstruct the dynamics of colonization which involved Corsica Island. Indeed, Wagner et al. [16], analyzing *S. fluviatilis* from Corsica, highlighted the importance of focusing studies on island populations due to the peculiarity and fragility of the island’s fish fauna, to infer the biogeographical dynamics of the populations that live there. These same authors also expressed hope for extending the geographical sampling to provide a more comprehensive scenario of *S. fluviatilis* in the Mediterranean area.

Focusing on Italy, *S. fluviatilis* is distributed in a discontinuous way, starting from the northern regions and continuing through the Tyrrhenian side, down to Campania, Sardinia, and the Sicily islands. Isolated populations have also been found in Calabria and on the Adriatic coasts of the peninsula [26,27]. Although the Italian peninsula hosts different populations of *S. fluviatilis* and the country is characterized by different (hydro)geographic boundaries that strongly shaped its ichthyofauna with the presence of several endemic species—64 taxa are considered native to inland waters of Italy [28] and among these at least 15 species are endemic [29]—no wide-range molecular studies have been carried out on this species in Italy to date. Populations of *S. fluviatilis* living on Sardinia Island deserve particular attention, as this species is one of the few putative native freshwater fish known for the island [30]. The following two problems are particularly serious for freshwater Sardinian fauna: the introduction of a high number of alien species, most of them perpetrated in the past three decades [30,31,32,33,34] and the freshwater habitat alteration, consisting mainly of the construction of numerous dams starting from the beginning of the last century. In such a context, the present study was aimed at filling a gap in genetic information on populations of *S. fluviatilis* from Italian areas that are not yet under investigation. Indeed, knowledge of the genetic structure of populations from north-west Italy and the Sardinian Island could help to fill in some gaps in the mosaic of *S. fluviatilis* genetic distribution in the central-western Mediterranean.

As a results, the goals of our paper were: (1) to depict the phylogeographic patterns of *S. fluviatilis* populations, sampled in Sardinia and north-west Italy (Liguria, Piedmont, and Lombardy) in order to shed light on the phylogeography of the populations now living in the main central-western Mediterranean islands and the north-west Italian mainland; (2) to compare the genetic structure of our samples with that of other Mediterranean regions to put them in a wider geographic framework; and (3) to make an attempt to further clarify the taxonomy of the species across its whole range of distribution by using different methods of species delimitation to evidence, if any, distinct evolutionary lineages within *S. fluviatilis* that deserve to be deeply investigated in the future.

Two portions of the mitochondrial Control Region and of the 16s ribosomal gene were used as markers. The use of the mtDNA, which is maternally inherited, is considered an appropriate choice to solve intraspecific taxonomic and phylogenetic issues and to depict evolutionary lineages [35,36,37]. Furthermore, because of its high mutational rate, mtDNA represents a practical tool to obtain preliminary inferences about the level of genetic variability among populations [38]. The high number of mitochondrial Control Region and 16s sequences that are present in the GenBank database for *S. fluviatilis* allowed us to compare the sequences obtained in the present study with many others from different geographic areas. In previous studies, the combined use of Control Region and 16s sequences has been demonstrated to be helpful in providing insights into the genetic structuring and phylogenetic history of *S. fluviatilis* populations [1,8,15,17].

## 2. Materials and Methods

### 2.1. Sample Collection

**Figure 1:**
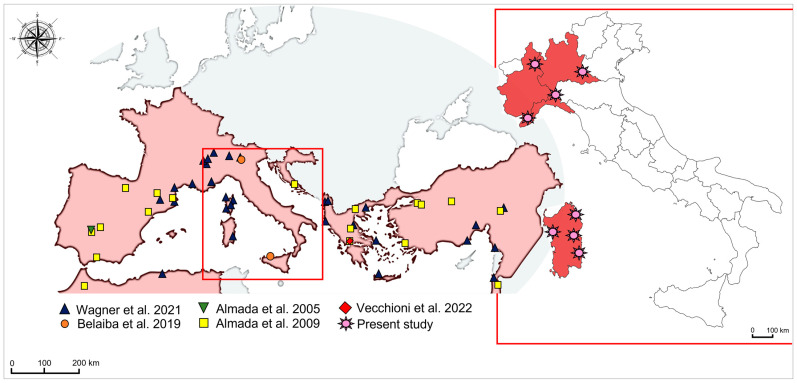
Map of samples’ collection sites. The map shows the geographical origin of the sequences isolated in the present study along with those from previous research [1,8,15,16,24].

A total of 103 individuals of *Salariopsis fluviatilis* were caught using an electric stunner from the freshwaters of 4 different Italian regions (Sardinia, Liguria, Piedmont and Lombardy) between February 2019 and March 2022 (Table S1 and Figure 1).

The protocol of sampling and analysis of the fish fauna of wadable lotic systems, provided by the Italian Higher Institute for Environmental Protection and Research (ISPRA) [39], was followed for the sampling collection in the present study. In accordance with this document (whose guidelines are compulsory in Italy), which requires that all electrically stunned fish must be collected, recorded and returned to the water, the individuals of *S. fluviatilis* analyzed in this study were caught using an electric stunner from freshwaters, subjected to a non-lethal sampling method by means of small tissue portion removal (fin-clips) and immediately transferred to a recovery tank before being released. Tissues were preserved in absolute ethanol and used to perform DNA extraction. The above reported sampling method was approved by the ethics committee of the University of Sassari (Prot n. 122 770 of 7 November 2022), and its researchers led the sample collection activities during the present study.

Sardinian samples were collected in three tributaries of the Flumendosa river (Accu terrale creek, Riu Pale creek, Sicaderba creek), all confluent in the Alto Flumendosa man-made reservoir (originated in the middle of the last century by a dam) (central-east Sardinia), the Rio Mannu di Scano Montiferro river (central-west Sardinia), and the Rio Mannu di Posada river (north-east Sardinia); Ligurian samples were collected in the Entella river (east Liguria) and in the Roja river (west Liguria); samples from Piedmont were collected in the San Giovanni river, in the San Bernardino river, and in the Strona di Omegna river; the sample from Lombardy was collected in the Tartaro Fuga creek, tributary of the Oglio river.

### 2.2. Implemented Molecular Analysis

Total genomic DNA was isolated from a portion of fin tissue using the Macherey-Nagel Nucleo Spin Tissue Kit (MACHEREY-NAGEL GmbH & Co. KG, Düren, Germany), following the supplier’s instructions. DNA solutions were quantified using the Nanodrop™ Lite Spectrophotometer (by Thermo Scientific; Waltham, MA, USA), which showed an average yield of 29 ng/µL. A portion of the mitochondrial Control Region and rRNA gene 16s were amplified by standard PCR using the following primers: CR-F (forward) (5′-CCACTAGCTCCCAAAGCTA-3′) and CR-R (reverse) (5′-CAGGACCAAGCTTTTGTGC-3′) [40]; 16s For (5′-CGCCTGTTTATCAAAAACAT-3′) and 16s Rev (5′-CCGGTCTGAACTCAGATCACGT-3′) [41]. Reactions were carried out in a total volume of 25 µL. On average, 10 ng of total genomic DNA were combined with 0.6 µM of each primer and one pellet of PuReTaq Ready-To-Go PCR beads (GE Healthcare, Wauwatosa, WI, USA) containing stabilizers, 4 ng of bovine serum albumin (BSA), deoxynucleotide triphosphates, 2.5 units of PuReTaq DNA polymerase, and reaction buffer. When a bead was reconstituted to a 25 µL final volume, the concentration of each dNTP and MgCl_2_ was set at 200 µM and 1.5 mM, respectively. PCRs were performed in a GeneAmp PCR System 9700 Thermal Cycler (Applied Biosystems, Waltham, MA, USA), programmed as follows: 1 cycle of 4 min at 94 °C, 35 cycles of 30 s at 94 °C, 30 s at 51 °C (for both Control Region and 16s gene), and 30 s at 72 °C. At the end, a post-treatment of 10 min at 72 °C and a final cooling at 4 °C were carried out. Both positive (high-quality DNA samples from the species *Micropterus salmoides*) and negative controls were used to test the effectiveness of the PCR protocols and the absence of possible contaminations. Electrophoresis was carried out on 2% agarose gels, prepared using 1× TAE buffer (Tris-Acetate-EDTA, pH 8.3) and stained with Gel Red Nucleic Acid Stain (Biotium Inc., Fremont, CA, USA). PCR products were purified by ExoSAP-IT (USB Corporation, Cleveland, OH, USA) and sequenced for forward and reverse strands (by means of the same primers used for PCR), using an external sequencing core service (Macrogen, Europe, Amsterdam, The Netherlands).

### 2.3. Phylogenetic and Phylogeographic Analyses

A total of 103 newly generated sequences was aligned using the package Clustal Omega [42] (available at https://www.ebi.ac.uk/Tools/msa/clustalo/ (accessed on 16 September 2022)) and deposited in GenBank (OP675744-OP675846 for the Control Region and OP653611-OP653713 for 16s).

The Control Region and 16s *S. fluviatilis* sequences obtained in the present study were concatenated to obtain a dataset as polymorphic as possible to infer on the phylogeographic relationships among populations from Sardinia and mainland northern Italy. Furthermore, with the aim of performing phylogenetic and molecular taxonomy analyses on the species on a wide Mediterranean context, the Control Region and the 16s sequences that were collected in the present study were also merged and aligned with those from other localities (Italy, Switzerland, Croatia, Albania, Greece, Israel, Morocco, Portugal, Spain, Turkey, Syria and Algeria) available on GenBank to date (last update 30 September 2022) (see Tables S2 and S3).

In the Control Region dataset, all the sequences available for the Mediterranean freshwater blennies were also included, i.e., *S. economidisi* (FJ465540, FJ465541, MZ026042, MZ026043, MZ026044) and *S. atlantica* (FJ465527, FJ465526). Furthermore, the marine species *Salaria basilisca* (MW555061) and *Salaria pavo* (MW555062) were used as outgroups. As well, in the 16s dataset, the sequences available for the Mediterranean freshwater blenny species were also included, i.e., *S. economidisi* (FJ465733, FJ465735) and *S. atlantica* (FJ465736, FJ465737). The marine species—*S. basilisca* (MH724820) and *S. pavo* (FJ465707)—were used as outgroups (see Table S3).

The genetic variation within the datasets was assessed estimating the number of polymorphic sites (S), number of haplotypes (H), haplotype diversity (h), and nucleotide diversity (π) using the software package DnaSP 6.12.03 (Barcelona, Spain) [43].

To test the reliability of the three datasets (i.e., Control Region, 16s and concatenated dataset) for taxonomic and phylogenetic analyses, the phylogenetic signal was checked by the software TREEPUZZLE (Wien, Austria) [44]: the likelihood-mapping analysis of 10,000 random quartets, performed for both genes was used and the general time reversible (GTR) was selected as the model of substitution (following Scarpa et al. [45,46]).

The best probabilistic model of sequence evolution was achieved using jModelTest 2.1.3 (Ballwin, MO, USA) [47], with a maximum likelihood optimized search by the Akaike (AIC) and Bayesian Information Criterion (BIC). The TPM3uf+I+G was suggested by the AIC and the HKY+G by the BIC as the best-fit model for the Control Region dataset, while the TPM2uf+G by AIC and the TPM2+G by BIC were selected for the 16s dataset. The pairwise genetic distances were estimated between populations using the software Mega 6.06 [48] with 1000 bootstrap replications. The correction according to the Kimura two-parameter model (K2P) [49] was applied (see Scarpa et al. [50] for methods).

To infer the genetic relationships among haplotypes and to detect the possible occurrence of discrete genetic clusters, a median-joining network [51] was constructed by means of the software Network 10.2.0.0 (www.fluxus-engineering.com) (accessed on 12 October 2022) (Colchester, UK). Transitions and transversions were equally weighted. Due to the absence of information about the possible appearance of retro-mutation events, the same weight (10) was assigned to each observed polymorphism.

Phylogenetic relationships were investigated on two datasets including all the sequences of Mediterranean blennies belonging to the genus *Salaria* from GenBank: they grouped 280 sequences for the Control Region (see Table S2) and 136 sequences for the 16s (see Table S3). Analyses were based on Bayesian Inference (BI) and performed by means of the software MrBayes 3.2.7 [52]. The BI was implemented specifying a partitioned model and setting as model parameters: NST = 6, rates = invgamma, ngammacat = 4. Two independent runs each consisting of four Metropolis-coupled Markov chain Monte Carlo (MCMC) chains (one cold and three heated chains) were run simultaneously for 5,000,000 generations, sampling trees every 1000 generations. The first 25% of the 10,000 sampled trees was then discarded as burn-in (see Scarpa et al. [50]). In order to assess the convergence of chains, it was checked that the Average Standard Deviation of Split Frequencies (ASDSF) approached 0 [52] and the Potential Scale Reduction Factor (PSRF) was approximately 1 [53]. Nodes with a percentage of posterior probability lower than 95% were considered not highly supported. The phylogenetic tree was visualized and edited using FigTree 1.4.0 (http://tree.bio.ed.ac.uk/software/figtree/) (accessed on 18 September 2022) (see Scarpa et al. [50]).

The taxonomic identity of each *S. fluviatilis* sequence was checked using five different methods of species delimitation. The GMYC (Generalized Mixed Yule Coalescent) model tests for a significant shift in the branching rate along an ultrametric species tree. The analysis was performed on the ultrametric species tree obtained from the Bayesian dating analyses by means of the SPLITS (SPecies LImits by Threshold Statistics) package [54] implemented in the R statistical environment (available at http://r-forge.rproject.org/projects/splits/)(accessed on 18 September 2022). Species entities were identified by means of the single threshold option, which uses a single threshold in order to specify the transition from between- to within-species branching (see Scarpa et al. [55]).

The PTP (Poisson Tree Processes) [56]) uses the number of substitutions to assess the speciation rate. Species delimitation was achieved by means of the PTP web server (available at http://species.h-its.org/ptp/) (accessed on 18 September 2022) on the phylogenetic species trees using default options and 500,000 MCMC generations. To test the reliability of results, each run was examined for convergence by visualizing the likelihood plot: if convergence occurred, the chain should stay at high likelihood locations during the run (see Scarpa et al. [55]).

The ABGD (Automatic Barcode Gap Discovery) [57] method is based on the K2P genetic distances [49]. The ABGD does not consider phylogenetic relationships within the dataset and works on sequences, identifying the barcode gap as the first significant gap beyond this limit and using it to partition the data [57]. Species were assessed by means of the ABGD online tool (available at http://wwwabi.snv.jussieu.fr/public/abgd/abgdweb.html) (accessed on 18 September 2022) using the default settings. The correct species estimate was selected, according to Puillandre et al. [57], using the gene specific priors for maximum divergence of intraspecific diversity, corresponding to *p* = 0.001 (see Scarpa et al. [55]).

The NDT (Nucleotide Divergence Threshold) method was used on each gene separately, by means of a script developed by Scarpa et al. [58], written in the R statistical environment (available at https://cran.rproject.org/) (accessed on 18 September 2022). The script partitions taxa into entities applying the fixed threshold of 2% obtained by Hebert et al. [59], using a pairwise Kimura two-parameter model (K2P) [49] genetic distances matrix (see Scarpa et al. [45]).

The principal coordinates analysis (PCoA) was performed using GenAlEX 6.5 [60] on a matrix of pairwise genetic distances corrected according to the Kimura two-parameter model (K2P), with the aim of identifying potential subgroups within the genetic clusters and to determine the dissimilarity represented by the genetic variation among sequences (see Tran Thi et al. [61]).

### 2.4. Estimation of Divergence Time Analyses

The software package Beast 1.10.4 [62] was used to estimate the divergence time for the clades evidenced by the Control Region phylogenetic tree, applying the substitution rate estimated on another blennioid species, *Tripterygium delaisi*, in a previous study by Koblmüller et al. [63]. Specifically, a 95% HPD (high density probability) interval of 1.1–6.67% per million years was fixed [16]. Site parameters Substitution Model = GTR; Bases Frequencies = Estimated; Site Heterogeneity Model = Gamma + Invariant Sites; Number of Gamma Categories = 4 have been set accordingly to the evolutionary models selected by jModeltest with the GTR model. For the molecular clock rate variation model, the lognormal uncorrelated relaxed clock was selected as it assumes independent rates on different branches. For the tree prior, the Yule prior process was applied to the speciation model. The priors for model parameters and statistics have been determined for calibrating the time-tree assuming the mutation rates per million years. Divergence times were estimated using a normal distribution with lower, central and upper values set according to the mutation rate per million years. Operator parameters have been fixed following the instructions of the user manual. Additionally, the application of the lognormal uncorrelated relaxed clock model gives an indication of the state of the clock-like data (measured by the ucld.stdev parameter). If the ucld.stdev parameter-estimate is close to 0, then the data is quite clock-like, and if it has an estimated value much greater than 1, then data exhibits very substantial rate heterogeneity among lineages. To obtain an effective sample size (ESS) greater than 200 for all the statistic parameters, a run of 200,000,000 generations was performed, sampling a tree every 20,000 generations following Scarpa et al. [50]. The software Tracer 1.6 (©2022 BEAST Developers. All rights reserved.) was used to view the resulting log file, with the aim of ensuring convergence of parameter values, to verify whether ESS values exceeded 200 and to estimate node ages [64]. Tree Annotator and FigTree were used for drawing and visualizing the time calibrated tree, following Scarpa et al. [50].

## 3. Results

A total of 103 sequences for both Control Region (302 bp) and 16s gene (547 bp) were obtained in the present study. Both datasets were merged to Genbank sequences (280 for the Control Region and 136 the 16s gene) and used to implement phylogeographic analyses.

All the newly generated sequences were identified as belonging to the species *Salariopsis fluviatilis* (sub *Salaria fluviatilis*) through Basic Local Alignment Search Tool (BLAST) analysis implemented in the GenBank nucleotide database (www.ncbi.nlm.nih.gov) (accessed on 24 August 2022) that showed a percentage of identity ranging from 97% to 100% for the Control Region and from 90% to 100% for the 16s gene.

In accordance, all methods of species delimitation suggest that the sequences isolated from Italian samples in the present study belong to the taxonomic entity of *S. fluviatilis* (see Table S4 for details). After reaching the correct taxonomic identification, further analyses were performed to infer the levels of genetic variation and genetic structuring among populations in Italy and other geographic areas.

### 3.1. Phylogeographic Relationships among New Samples from North-West Italy and Sardinia Island

Among the 103 sequences obtained in the present study for the Control Region in samples from Liguria, Piedmont, Lombardy and Sardinia, 16 polymorphic sites resulting in 14 haplotypes were found (see Table 1).

The overall level of haplotype diversity found for samples from Sardinia was very low with only 4 similar haplotypes found out of 47 sequences and a reduced nucleotide diversity (π = 0.00046). For the two samples from Lombardy only one haplotype was retrieved, while higher levels of divergence were found for samples from Liguria and Piedmont with 8 and 4 haplotypes, respectively.

Regarding the 103 sequences obtained in the present study for the 16s gene, low levels of divergence were found for each region with a total of 7 polymorphic sites resulting in 8 haplotypes (see Table 1). Samples from Liguria, Piedmont and Lombardy showed the lowest levels of diversity.

With the aim to obtain a dataset including the level of polymorphism and informativeness as high as possible, these two datasets of sequences (Control Region and 16s) were concatenated to obtain a longer mitochondrial fragment, which was 849 bp long, to perform phylogeographic analyses. In this concatenated dataset, 23 polymorphic sites were found, resulting in 20 haplotypes and moderately low levels of haplotype and nucleotide diversity (see Table 1). A similar level of diversity was found for all the regions analyzed (with the exception of Lombardy). In accordance with the trend previously reported for the 16s datasets, the highest level of divergence was found in Sardinia; however, in this island 6 polymorphic sites were found, resulting in 7 very similar haplotypes, as suggested by the lowest (with the exception of the two samples from Lombardy) rate of nucleotide diversity (π = 0.00038) (see Table 1).

**Figure 2:**
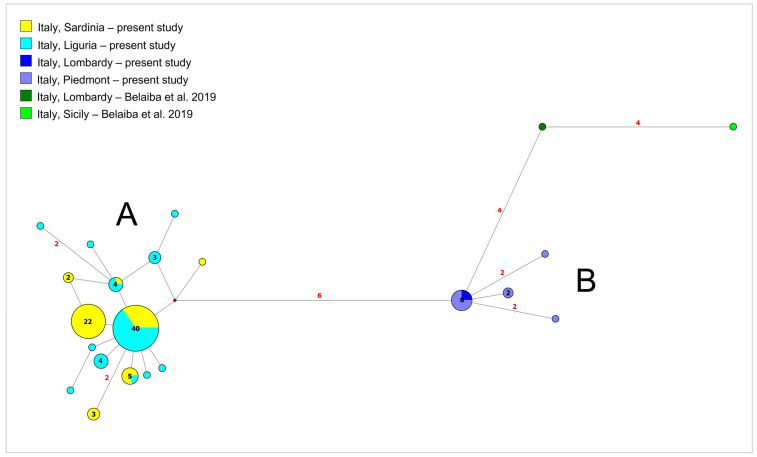
Median-joining network analysis. The network includes all the Italian sequences (from the present study and also from a previous study [15]) for the Control Region and 16s (merged in a concatenated dataset). Clusters A and B are described in the text. The number of mutations between sequences that are greater than *n* = 1 are reported on network branches. The number of individuals showing the same haplotype that is greater than *n* = 1 is reported inside the spots. Belaiba et al., 2019 in the legend corresponds to the reference [15].

The network analysis was performed on a concatenated (Control Region and 16s) dataset which included samples from northern Italy and Sardinia (see Figure 2) from the present study. In order to obtain a scenario as complete as possible of the phylogeography of *S. fluviatilis* in Italy, the two Italian sequences (from the Lombard side of Lake Garda and Sicily) which had been previous published [15] were included in the dataset. The analysis took into account gaps and redundant polymorphic bases and evidenced the occurrence of a genetic structuring between two main groups of sequences (Groups A and B) which diverged for 6 point mutations among each other.

Group A included individuals from Sardinia and Liguria with the occurrence of two main star-like shapes involving the two areas. In particular, among samples from Liguria, a high level of variability was witnessed by the number of different haplotypes that are present in this area with a strong founder effect, as suggested by the occurrence of a very common central haplotype (that is also shared by 29.8% of Sardinian individuals), which is surrounded by several derived haplotypes diverging for a few mutations. The haplotypes that likely originated from the lineages most common in Liguria, are private to single Ligurian individuals (with one exception for one haplotype shared by three individuals from Sardinia) or shared among Sardinian and Ligurian individuals. In only two cases, haplotypes likely derived from the Ligurian ancestor lineage are exclusive to Sardinian individuals. In particular, one of these two latter cases corresponds to the most common haplotype among Sardinian individuals, which was found in 46.8% of specimens collected and is private to Sardinia. In particular, only one haplotype isolated in the north-east of the island (Rio Mannu di Lodè river), which is exclusive to a single individual, could have not necessarily originated from the most common Ligurian and Sardinian founder lineages.

Group B included samples of Piedmont and Lombardy from the present study with a marked founder effect suggested by the occurrence of a star-like shape. The two identical sequences found in Lombardy correspond to the most common haplotype occurring in Piedmont. Two out of three derived haplotypes were private of single Piedmontese individuals. The sequence from Lake Garda (Lombardy) from a previous study [15] was included within this group diverging for four points mutations from the most common haplotype. Additionally, the sequences from Sicily from a previous study [15] were included within this group, diverging for four points mutations from the sequence from Lake Garda.

**Figure 3:**
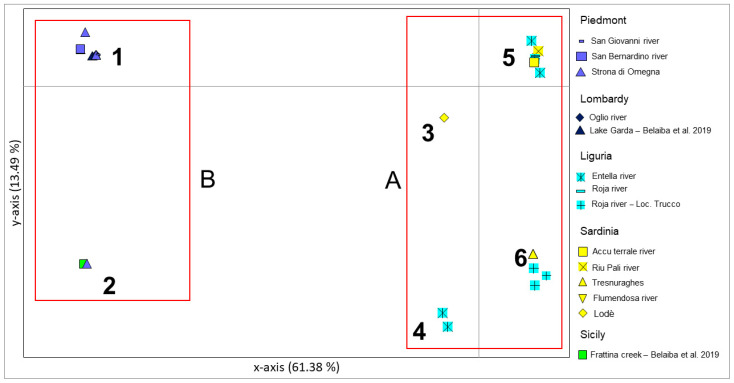
Principal coordinates analysis performed on Italian sequences from the present study and also from a previous study [15]. Bidimensional plot shows the genetic differentiation among specimens due to the nucleotide substitutions per site found in the dataset. Belaiba et al., 2019 in the legend corresponds to the reference [15].

In the PCoA graph (see Figure 3 and Table S5) the 61.38% of variability was explained by *x*-axis and *y*-axis and accounted for only 13.49%.

Results were consistent with the genetic structuring evidenced by network analysis, suggesting a divergence along the *x*-axis between two main groups (Groups A and B). Group A included individuals from Liguria and Sardinia with an internal, although poorly supported, sub-structuring. Indeed, an eastern Sardinian sample from the Rio Mannu di Lodè river (subgroup 3 in Figure 3) and a group of four samples from east Liguria (subgroup 4 in Figure 3) collected in the Entella river, slightly diverged from the remaining samples represented by two different clusters of sequences. The first of these two clusters (subgroup 6 in Figure 3) included only few samples from west Liguria (five individuals from Roja river), three individuals from central-west Sardinia: two from Rio Mannu di Scano Montiferrro river and a further sample from the Rio Mannu di Lodè river. The second cluster (subgroup 5 in Figure 3) of sequences was the larger in the graph and included individuals from all the Sardinian and Ligurian sampling sites.

Group B of PCoA included all samples from Piedmont and Lombardy in a unique cluster with the only exception of one Piedmontese sample (subgroup 2 in Figure 3) from the Strona di Omegna River in the north-east of Piedmont, which diverged from the main group (subgroup 1 in Figure 3) on the *y*-axis.

### 3.2. Phylogenetic Reconstruction and Species Delimitation Based on Control Region

Both phylogenetic and species delimitation analyses were based on the Control Region dataset that showed a strong phylogenetic signal (see Figure S1a). In particular, either the reduced number of specimens available on GenBank for which sequences of Control Region and 16s were present, and the low phylogenetic signal obtained for 16s (see Figure S1b) prevented us from applying those analyses to a reliable concatenated dataset in terms of a phylogenetic signal (see Figure S1c). Indeed, the test of the Likelihood Map disassembled the dataset in quartets, that represent the smallest set of taxa for which more than one unrooted tree topology exists [65]. The quartet puzzling works on groups of four sequences, in order to obtain a map that allows for understanding whether data are reliable for phylogenetic and taxonomic inferences. The most important information in the map is given by the percentage of star-like trees (which represent the area of the unsolved trees), which, when higher than 30%, suggests that the dataset is not reliable for analyses, due to noisy data; alignment errors; recombination events; not enough informative sites [66]; or inadequate taxonomic coverage (see Scarpa and co-authors [67]). The Control Region dataset showed a percentage of points in the network-like areas of 23.5% (see Figure S1a) and the 16s dataset 92.2% (see Figure S1b). As expected, the high level of noisy data and the low level of information sites in the 16s dataset also affected the concatenated dataset, which showed in the network-like areas a percentage of 79.7% (see Figure S1c). For these reasons, both 16s and concatenated datasets were considered as not reliable for phylogenetic and taxonomic purposes and were not used for phylogenetic analyses in the present study.

**Figure 4:**
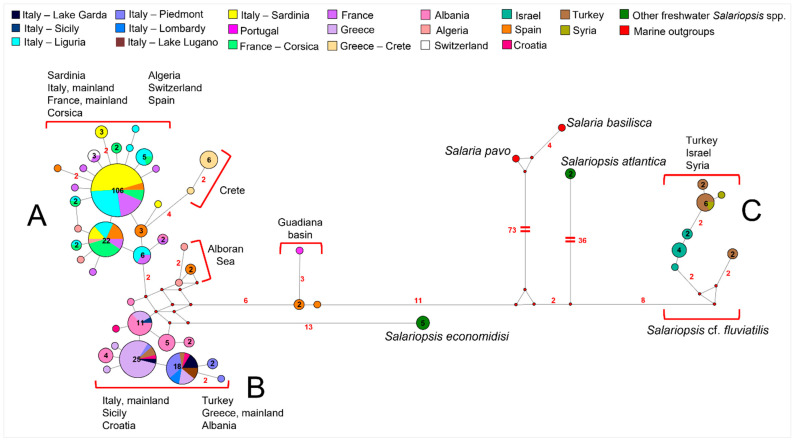
Median-joining network analysis performed on the whole Control Region dataset. Clusters A, B and C are described in the text. The number of mutations between sequences that are greater than *n* = 1 are reported on network branches. Additionally, the number of individuals showing the same haplotype that is greater than *n* = 1 is reported inside the spot.

The network analysis performed on the Control Region dataset, including all the sequences available for the Mediterranean freshwater blennies (see Figure 4 and Table S2), showed the presence of three main clusters: cluster A, cluster B, and cluster C.

Cluster A included all the Sardinian and Ligurian samples, together with all the sequences from mainland France, Corsica, Algeria, Switzerland, and almost all from Spain. Interestingly, sequences from Crete showed only two haplotypes which resulted endemic of the island and quite divergent from the others (4 to 6 mutational steps apart from cluster A).

Cluster B was representative of northern Italy (Lake Garda, Piedmont, and Lombardy, excluding Liguria) and Sicily, along with the eastern part of continental Europe (Croatia, Albania, Greece mainland, and western Turkey coasts on the Aegean and the Marmara seas). Furthermore, three sequences from Spain and one from Portugal from the basin of the Guadiana River, which flows into Atlantic Ocean, set 6 to 9 point mutations apart from clusters A and B. Interestingly, a group of two Spanish and two Algerian sequences, all collected throughout a geographic area facing the Alboran Sea, sets in the network on a separate position between clusters A and B diverging for 4 point-mutations from each of them.

Cluster C grouped the sequences from southern Turkey (but also one from Ilica River in the north) that Belaiba et al. [15] identified as *S.* cf. *fluviatilis*, along with sequences from Syria and Israel. Within this cluster, all the haplotypes found in Israel were exclusive to this area, while a common haplotype was shared between Turkey and Syria. Cluster C diverged from the previous reported Guadiana River group for 22 to 29 point mutations.

The sequences of *S. economidisi*, endemic of Lake Trichonis in Greece, sets close to cluster B, 13 point-mutations apart. The two sequences representing *S. atlantica*, which was described in Morocco, were found 44 mutational steps far from cluster C, 50 from the Guadiana River group, and approximately 60 from clusters A and B.

The phylogenetic tree (see Supplementary Figure S2) showed the presence of a large monophyletic clade, including all the Italian sequences belonging to *S. fluviatilis* together with the Mediterranean sequences taken from GenBank, and it split into a dichotomy characterized by two main clusters (clusters 1 and 2). The first cluster (cluster 1) showed a polytomic clade, including almost all *S. fluviatilis* sequences, and its sister clade representative of *S. economidisi* (represented by two sequences).

The large polytomy of *S. fluviatilis* grouped all the Italian sequences along with those from eastern Europe, mainland France, Corsica, Spain, Portugal, Switzerland, and Algeria. It includes some internal well-supported sub-clusters; among them, the three most relevant groups: (i) three Spanish and one Portuguese sequences corresponding to the Guadiana River group evidenced by network analysis, (ii) two Spanish and two Algerian sequences, and (iii) seven sequences from Crete. These three well-supported internal subgroups were also found by network analysis. The second cluster (cluster 2) of the tree included the Turkish, Syrian, and Israeli sequences, which grouped together in the cluster C of network analysis. Accordingly, with network analysis, an internal sub-structuring, consistent with the geographic distribution of samples, was also found in cluster 2 of the phylogenetic tree.

Species delimitation methods outcomes (see Table S4) showed some discrepancies among each other, consistent with the characteristics of methods (see the Section 2 for details). Focusing on *S. fluviatilis*/*S.* cf. *fluviatilis*, the NDT and the ABGD methods converged in recognizing the same three entities. The first entity comprises Italian, French, Swiss, Algerian, Balkan and almost all Spanish sequences. The second entity includes the Turkish, Syrian and Israeli sequences corresponding to the group (including *S.* cf. *fluviatilis*) already retrieved by previous analyses. The third includes the Portuguese and Spanish sequences from the Guadiana River basin (in accordance with results already obtained by Wagner et al. [16]).

On the other hand, the PTP method detects four entities, partially converging with the NDT and ABGD but grouping the Guadiana River entity within the “European” entity, and splitting the group of Turkish, Syrian, and Israeli sequences into three separate entities. The GMYC methods found a unique entity for all the sequences belonging to the genus *Salariopsis* included in the datasets.

**Figure 5:**
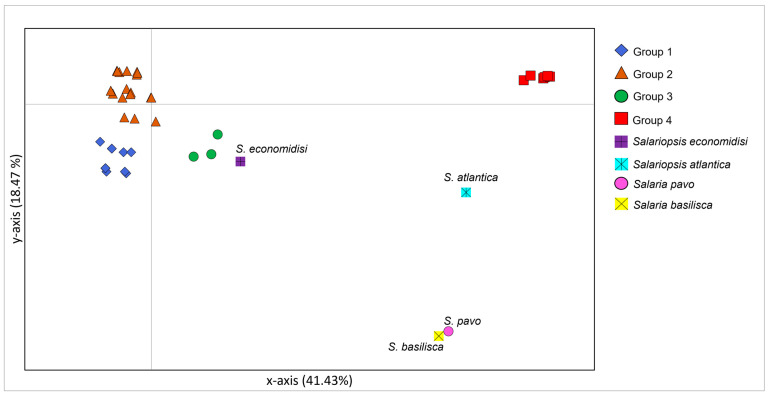
Principal coordinates analysis performed on the whole Control Region dataset. Bidimensional plot shows the genetic differentiation among specimens due to the nucleotide substitutions per site found in the dataset.

In the PCoA analysis (Figure 5 and Table S6), the 41.43% of variability was explained by *x*-axis while *y*-axis accounted for 18.47%.

Results are consistent with the differences among *S. fluviatilis* Mediterranean populations evidenced by previous analyses, distinguishing four groups: (a) Group 1, which includes sequences from Greece, northern Italy (apart from Liguria), Sicily, Croatia, Albania, and some sequences from the western coasts of Turkey facing the Aegean and the Marmara seas; (b) Group 2, which includes sequences from Sardinia, Liguria, Spain, mainland France, Corsica, Switzerland, and Algeria; (c) Group 3, which includes the Guadiana River sequences from Spain and Portugal; and (d) Group 4, which includes sequences from Turkey that Belaiba et al. [15] identified as *S.* cf. *fluviatilis*, Israel and Syria. It is important to note that group 1 and group 2 diverged only on the *x*-axis which accounts for only 18.47% of variation suggesting that, according to Network analysis, these two groups are structured among each other but with a weak genetic divergence.

*Salariopsis economidisi* and *S. atlantica*, and the two marine outgroups, *S. pavo* and *S. basilisca*, set in the plot apart from the four *S. fluviatilis*/*S.* cf. *fluviatilis* groups.

The time calibrated tree (Figure S3), obtained by the software Beast, was consistent with the Bayesian phylogenetic tree (see Supplementary Figure S2). The common ancestor to all Mediterranean *Salariopsis* species dates back to 990 kya. The common ancestor of the group of sequences, including *S. fluviatilis*, *S.* cf. *fluviatilis* and *S. economidisi* (see SEGR2_FJ465541_1 and SEGR1_FJ465540_1 in the tree) dates back to approximately 800 kya. *Salariopsis atlantica* (see SAMA2_FJ465526 and SAMA1_FJ465527 in the tree), which originated in 390 kya, sets in the phylogenetic tree as an external clade to the cluster including *S. fluviatilis*, *S.* cf. *fluviatilis* and *S. economidisi.*

The large group of sequences of *S. fluviatilis*, which includes Italian, Eastern European, French, Corsican, Spanish, Portuguese, Swiss, and Algerian individuals, along with the sequences belonging *S. economidisi*, differentiated approximately 610 kya. Within this group, the common ancestor to *S. fluviatilis* diverged approximately 575 kya, while the common ancestor of *S*. *economidisi* dates back to approximately 130 kya. Within the large clade of *S. fluviatilis*, it was possible to obtain a molecular dating for the three well-supported groups of sequences reported in the previous analyses. One group corresponds to the sequences from the Guadiana River basin and dates back to approximately 180 kya (with two internal subgroups that originated approximately 40 and 20 kya, respectively), another group of Spanish and Algerian sequences from the Alboran Sea area, dates back to approximately 150 kya (with two internal subgroups that originated approximately 120 and 30 kya, respectively), and the last group, including Cretan sequences, dates back to approximately 210 kya (with an internal subgroup that originated approximately 80 kya).

The group of sequences, including the Turkish, Syrian and Israeli *S.* cf. *fluviatilis* sequences, date back to approximately 630 kya.

### 3.3. Phylogenetic Reconstruction Based on 16s Gene

The low level of genetic variation found for the 16s dataset (see Table 1) may have affected the values obtained from the phylogenetic signal test that suggested a lower resolution for phylogenetic and taxonomic analyses if performed. The scarce informativeness of the 16s fragment in this study may be explained considering that it is a mitochondrial gene highly conserved, therefore it generally shows lower levels of genetic differentiation [67] and a slower evolutionary rate if compared to the Control Region.

However, even taking into account the low level of polymorphism and the reduced number of available sequences for the 16s dataset, the network analysis (Figure S4) was consistent and corroborated results obtained from the Control Region, with a few discrepancies. Indeed, results obtained evidenced the occurrence of two main clusters of sequences (A and B) for *S. fluviatilis* and two further small clusters of sequences for (1) the Guadiana River basin and (2) western Turkey (*S.* cf. *fluviatilis*) with Israel, respectively. Cluster A includes only sequences from the Italian regions of Piedmont, Lombardy, Liguria, and Sardinia, while cluster B includes sequences from Italy (only from Lake Garda in the north of the peninsula and Sicily Island), Croatia, Spain, Greece, and Turkey (in the West of the country). All the 16s sequences obtained in the present study were included within cluster A.

## 4. Discussion

The present study represents the first insight into the genetic variability of the Italian populations of *Salariopsis fluviatilis*: phylogeographic and phylogenetic analyses were performed including a high number of Italian specimens from the north-west regions of the peninsula (Liguria, Piedmont, and Lombardy) and the Mediterranean island of Sardinia, which represents an area so far neglected by molecular studies focused on this species. Moreover, molecular dating based on the present dataset, which covers most of the range of distribution of this species, gave us the possibility to put in an updated time range of the evolutionary patterns previously described for the new genus, *Salariopsis* [15,16]. Finally, the results obtained in the present study, which are fully supported by the application of five methods of species delimitation, corroborated previous hypotheses on the taxonomic status of some populations of Mediterranean freshwater blennies [15,16].

The mitochondrial DNA showed to be a suitable tool to illustrate phylogenetic and phylogeographic relationships among *S. fluviatilis* populations and to infer the taxonomic status of this species. In particular, the use of the mitochondrial Control Region has been effective in identifying genetically divergent groups within the species.

### 4.1. Genetic Structuring and Phylogeographic Patterns of Salariopsis fluviatilis in Italy

Our research evidenced a well-supported genetic structuring among Italian *S. fluviatilis* populations. Indeed, the group that includes Ligurian and Sardinian samples is strongly divergent from the group formed by samples from Piedmont and individuals from Lombardy and Sicily. The genetic structuring between *S. fluviatilis* from Liguria and Piedmont, which do not share common haplotypes among each other, may be ascribed to the geographic isolation between these flanking geographical areas. Indeed, the genetic pattern observed here can be explained considering that the presence in the Ligurian region of boundaries, represented by the Alps ridge in continuity with the Apennines, hindered the Ligurian individuals’ colonization of the northernmost Italian regions. This scenario is known for other species of Italian freshwater fishes as well, for instance, within the genus *Telestes*, Stefani et al. [68] reported the presence of two main mitochondrial clades within the Italian evolutionary lineage, which can be strongly traced geographically to the two biogeographical districts, Tuscan-Lazio, and Po Valley-Venetian, which are actually separated by the Ligurian Alps-Northern central Apennines system. As well, regarding the genus *Squalius*, the same barrier secretes *S. squalus* in the basins afferent to the Po River drainage network and *S. lucumonis* in the Tyrrhenian [69,70,71,72]. The pivotal role of the Appennines as biogeographic barrier for freshwater taxa has been evidenced in other vertebrates other than fish, such as in the European pond turtle (*Emys orbicularis*) (Testudines: Emydidae) [73] and also in invertebrate species, such as in the *Unio* spp. (Bivalvia: Unionidae) [74].

Worth noting, the sole Sicilian individual present in our dataset, from Belaiba et al. [15], grouped with the genetic cluster that also includes *S. fluviatilis* from Piedmont and Lombardy. This is an interesting finding, as it does not support the role ascribed to *S. fluviatilis* as a dispersal vector for the glochidia of the mussel of the genus *Unio* from Sicily to Sardinia or *vice versa*, suggested by Marrone and co-authors [74]. At the present state of the art, the absence of a genetic similarity between Sardinian and Sicilian populations would lead to rejection of this hypothesis, at least until samples that come from watercourses that flow into the Tyrrhenian Sea are analyzed. Indeed, the Sicilian individual was collected in the Frattina creek, a tributary of the Belice river, which flows into the Sicilian Sea, at the edge of the northern Siculo-Tunisian Strait. It is a biogeographic area separating the west and east Mediterranean in which different marine animals, with different dispersal capabilities, are anyway genetically separated from their Tyrrhenian counterpart (see [75,76,77,78] and references therein). Thus, to get a correct sight of the biogeographic position of Sicilian *S. fluviatilis*, a higher number of individuals, from different sites in Sicily, should be analyzed. A wider sampling campaign would also allow for enlarging knowledge on the genetic distribution of this island populations.

The genetic relationship that was found in the present study between samples of *S. fluviatilis* from Piedmont and Lombardy can be the result of movements of individuals from the Balkans through the Po river paleo-drainage that during the last glacial maximum included some Balkan tributaries. Indeed, the Balkan Peninsula was one of the main glacial refugia during the Pleistocene period, probably acting as a crossroad of different developmental processes [79]. Furthermore, our results demonstrated that, among Italian samples, northern populations showed the highest level of genetic variation. In contrast, Sardinian individuals showed several shared haplotypes with Ligurian populations, along with a few further private linages, which likely derived from Ligurian ancestors/founders.

### 4.2. Reconstruction of Phylogeography of Salariopsis fluviatilis in the Sardinia Island

Overall, a generally low level of genetic variation among the *S. fluviatilis* samples analyzed here has been found in populations from Sardinia. As expected, this finding reflects the typical evolutionary model of species dispersal on an island. Taking as a model the migration patterns reported for the *Salmo trutta* lineages [80,81,82,83], the following scenario could be invoked to explain our results concerning Sardinian populations of the species: the current distribution pattern of *S. fluviatilis* may be linked to the glaciation episodes that occurred during the Quaternary. Indeed, the climatic oscillations throughout this geological event may have promoted the migration of some individuals of *S. fluviatilis* (see Sanz et al. [84]) from an ancient dispersal center, identifiable in the Iberian Peninsula, as suggested by both the high level of genetic variability and the occurrence of several divergent mitochondrial lineages in that geographic area. Therefore, the spreading of *S. fluviatilis* might have reached Sardinia, following a stepping-stone migration model, starting from the Iberian Peninsula, and passing through Liguria and Corsica. In this context, it is worth mentioning that the island of Sardinia was alternately connected by land with the Corsica Island from the Miocene up to Pleistocene, and during the glaciation acmes, the distance between facing coasts of Corsica and the mainland substantially reduced, probably favoring some adults dispersal by sea. This possibility should be taken into consideration as Plaut [10] demonstrated that *S. fluviatilis* can survive and osmoregulate in seawater for a period of at least three months.

Due to low sea levels during Quaternary climatic oscillations, Sardinia may have maintained contact with the mainland via the Island of Elba, and during the Last Glacial Maximum (21 kya), connections between Corsica (a possible refuge area for *S. fluviatilis* during the late Pleistocene) and the Sardinian islands were renewed [16,25]. Consequently, according to a model proposed for the Sardinian populations of the brackish water fish *Syngnathus abaster* [41], the land bridges naturally created in the Quaternary between Tuscany and the islands of Elba, Corsica, and Sardinia might have facilitated the occurrence of gene flow between the continental populations of *S. fluviatilis* and those of the islands [85].

The generally low level of genetic differentiation among the Sardinian samples evidenced in the present study would represent the typical effect of genetic drift, which acts as an evolutionary force by means of the founder effect. Indeed a few lineages, also common in Liguria, might have reached Sardinia, becoming the ancestors of modern populations. According to this model, the frequency of founder lineages grows quickly in the areas recently colonized and new, poorly divergent and private haplotypes arise from the first strains that arrived.

A model of a stepping-stone for the Sardinian freshwater species and the genetic structuring between flanking Italian regions separated by the Alps could be taken into account to explain the results obtained by Wagner et al. [16], which demonstrated that haplotypes from Corsican rivers diverged from those found in the Italian Alpine lakes, instead grouping with those from rivers of various localities in France and northern Spain. These authors reported a genetic similarity of Corsican samples not only with French and Spanish populations but also with the very few individuals from Sardinia they analyzed. This suggests that a brackish bridge between Italy and Corsica during the ice melting following a glacial period could have prompted gene flow between the island and mainland populations [25,86]. Genetic similarity between samples from Corsica and those from the southern part of mainland France was also found by Laporte et al. [25] based on nuclear markers. In this context, Wagner et al. [16], claim that a limited taxon and geographical sampling prevented previous studies from providing a comprehensive picture of genetic and biogeographic relationships among and within major freshwater *Salariopsis* lineages. The first data from Ligurian populations obtained in the present study shed further light on the populations of Sardinia and Corsica Islands.

### 4.3. Uprising of Distinct Taxonomic Entities within Salariopsis fluviatilis

The large number of *S. fluviatilis* sequences obtained in this study from previously unexplored geographic regions provided new insights into the taxonomic status of this freshwater blenny in its whole distribution area. Indeed, the analysis of a large set of Control Region sequences from the whole range of distribution of the Mediterranean *Salariopsis* species allowed us to evidence the occurrence, and the temporal origin, of different taxonomic units within the genus *Salariopsis* in the Mediterranean freshwaters.

Remarkably, the molecular dating estimates obtained in the present study predate those provided by Wagner et al. [16] for the same (when possible) groups of populations. This finding may be a consequence of both the higher numbers of Italian sequences used in the present study and the different datasets used for molecular dating that might account for the discrepancies between the two studies. Furthermore, although we used the same substitution rate as Wagner et al. [16], which was based on the mitochondrial Control Region of the blennioid *Tripterygium delaisi* [63], such a substitution rate was here applied to a dataset including only mitochondrial sequences (Control Region), whereas Wagner et al. [16] used this substitution rate to perform a molecular dating based on a concatenated dataset, including both the mitochondrial Control Region and the nuclear first intron of the S7 ribosomal protein gene sequences, which has a notably slower evolutionary rate.

Wagner et al. [16] and Belaiba et al. [15] evidenced the presence of genetic divergence of *S. fluviatilis* populations from the eastern Mediterranean region and suggested that this group of individuals should be considered as *Salariopsis* cf. *fluviatilis*. Accordingly, results obtained in the present study show the occurrence of (1) genetic differentiation between two main groups of *S. fluviatilis* populations within the Mediterranean basin; (2) a group of populations from the Middle East represented by individuals from southern Turkey (but also one from the Ilica river in the north of the country), Israel and Syria which corresponds to *S.* cf. *fluviatilis* in accordance with Belaiba et al. [15]; and (3) a group of sequences of Iberian individuals from the basin of the Guadiana River which flows into the Atlantic Ocean.

Within the Mediterranean clade of *S. fluviatilis*, sequences from Italy (with the regions of Piedmont, Lombardy, and Sicily Island), Greece, Croatia, Albania, and part of Turkey (western coasts areas on the Aegean and Marmara seas) are representative of the North Oriental genetic cluster, whereas sequences from Spain, Italy (with Liguria and the Sardinia Island), Algeria, France mainland, and the Corsica Island represent the Occidental genetic cluster. Interestingly, individuals from the basin of the Guadiana River, which is the only investigated freshwater basin whose tributaries flow in the Atlantic, are slightly differentiated by the Occidental genetic cluster, suggesting an incipient genetic divergence of this population from the Mediterranean clade of *S. fluviatilis*.

The tolerance of freshwater blennies, and *S. fluviatilis* too [10], to a wide range of salinities might have favored the evolution from marine to freshwater forms, going through brackish, oligosaline and freshwater conditions. This might have encouraged the appearance of multiple discernible forms [16]. In particular, taking into consideration that the Guadiana genetic cluster is quite recent, according to the hypothesis proposed by Perdices et al. [7], Almada et al. [8] and Laporte et al. [9], it could have originated during the Pleistocene post-glacial event, between the end of Mindel and the beginning of Würm glaciations, from ancestors that were able to disperse via marine environments through adults dispersal. As an alternative hypothesis, we should take into consideration that *S. fluviatilis* colonized the upper Guadiana basin starting from river catches of the Júcar basin, as likely happened to the strictly freshwater fish *Luciobarbus guiraonis* (Cypriniformes: Cyprinidae: Barbinae) [87,88]. Nonetheless, at the present state of knowledge, the possibility of dispersal through marine environments is equally plausible, due to the high tolerance to the seawater osmolality of *S. fluviatilis*, at least for some months [10].

Furthermore, in accordance with Wagner et al. [16], within the genetic variation of *S. fluviatilis*, our results also evidenced the genetic divergence of the group of a few Spanish and Algerian sequences from the area of the Alboran Sea, and the group of sequences from Crete Island, which likely originated in the interglacial period between the Mindel and the Würm glaciations.

Interestingly, the *S. fluviatilis* population of Crete may be regarded as a peripheral isolate (sensu Frey, [89]), namely, a genetically isolated yet persistent population on the margin of the species’ existing range. Indeed, populations of *S. fluviatilis* may have diverged in Crete more than would be predicted from the degree of variation of the other Mediterranean populations, due to the very far distance of this island from the Mediterranean mainland coastline.

A similar phenomenon of both partial geographic isolation and local adaptation that prompt high genetic differentiation may also be invoked for the westernmost populations of *S. fluviatilis*, which live at the edge of the distribution of the species. The Alboran Sea can be considered a geographic area where surface marine circulation patterns may directly shape the genetic variation of marine organisms, and indirectly act on the genetic variation of brackish and freshwater species, thus producing a genetic divergence with other Mediterranean populations (e.g., Casu et al. [76]).

Interestingly, within the taxonomic entity *S. fluviatilis sensu stricto*, the geographic isolation among populations and the reduced gene flow prompted the genetic divergence among Occidental and Oriental groups of populations. In such a context, natural fragmentation or secondary contact between populations that were separated in the past but are currently present in adjacent areas may have further helped to generate the genetic differentiation observed between Liguria and Piedmont in northern Italy. However, such a divergence is not enough to be completely retrieved by species delimitation methods as a trace of incipient speciation, but it is suggestive of a relevant trend of ongoing genetic divergence.

Based on the divergence times estimated for the genetic clusters retrieved in the present study, it is possibly suggesting that the common ancestor to the Mediterranean species of *Salariopsis* originated approximately 1 million of years ago, before the beginning of Pleistocene glaciations. Then, repeated glacial fluctuations may have contributed to shaping the genetic divergence among fragmented populations of freshwater blennies, which were substantiated by speciation events in the genus *Salariopsis*. In such a context, the current distribution of *S. fluviatilis* and *S.* cf. *fluviatilis* lineages is the consequence of the Pleistocene post-glacial events that occurred between the end of Günz and the beginning of Mindel glaciations and that copious events of deglaciation may have led the structure of *S. fluviatilis* populations to be further shaped by the passage through different levels of salinity across river networks [25] and Pleistocene glacial fluctuations [16].

It is reasonable to consider the possibility that the evolutionary stages evidenced in the present study led *S. fluviatilis* to develop into a species complex as a consequence of past geological phenomena and present geographical boundaries. In this context, it is interesting to note that *S. fluviatilis* is separated from *S*. cf. *fluviatilis* by a number of mutations (23) that is greater than the number of mutational steps that separates it from the species *S. economidisi* (15), which is endemic to the western Greek Lake Trichonis [2]. Indeed, according to our results, which are based on the reduced number of sequences available on GenBank for this latter species, *S. economidisi* originated at the end of the interglacial period between the Mindel and Würm glaciations being likely contemporaneous with the Guadiana River taxonomic unit and to Alboran Sea and Crete Island genetic clusters.

*Salariopsis fluviatilis*, *S. economidisi* and the Guadiana River taxonomic unit share a unique common ancestor that likely originated during the Günz glaciation and that is contemporaneous with the ancestor of *S.* cf. *fluviatilis*. In accordance with Perdices et al. [7] and Neat et al. [6], these two ancient and contemporaneous ancestors might have belonged to one of the marine blennies species that differentiated in freshwater courses during Pleistocene glacial fluctuations and colonized the surrounding areas, which are interconnected by a large network of tributaries.

For this reason, at the current state of knowledge, the *S.* cf. *fluviatilis* taxonomic entity could be representative of a new species, distinct from *S. fluviatilis*, as *S. economidisi* and *S. atlantica*, which is pending a formal morphological description and new genetic analyses. In this context, it is interesting to note that *S.* cf. *fluviatilis* shows a genetic similarity higher with the species *S. atlantica*, which is endemic to the Seboui river basin in Morocco [17], rather than with the Algerian sequences of *S. fluviatilis sensu strictu*. This finding suggests that the Mediterranean area of North Africa could be inhabited by different freshwater species belonging to the genus *Salariopsis*, which may be characterized by relevant levels of genetic structuring so far undetected. What is reported here is in accordance with the phylogeographical patterns observed in other Maghrebian freshwater species (see, e.g., [90] and references therein). A similar trend was also hypothesized for the North African populations of the brackish water fish *Syngnathus abaster*, whose northern Tunisian individuals showed mitochondrial lineages belonging to either the westernmost Mediterranean genetic cluster or the Tunisian [41].

Interestingly, the genetic divergence found for the Guadiana River taxonomic entity and also for the two divergent clusters, found within *S. fluviatilis*, for the Alboran Sea area and Crete Island, may be the result of the genesis and consequent isolation of glacial refugia during the last glacial maximum (Würm), which has promoted genetic exchanges among river basins.

Overall, in the present study, the similar temporal estimates obtained by molecular dating for the origin of (1) *S. fluviatilis* and *S*. cf. *fluviatilis* (approximately 600 kya each) and of (2) the clusters of *S. economidisi*, Guadiana River, Alboran Sea, and Crete Island (on average 135 kya) suggest the contemporaneous origin of these taxonomic entities. This finding supports the occurrence of similar evolutionary rates for these groups produced by the perfect timing of the mitochondrial DNA molecular clock for all Mediterranean freshwater blennies whose common ancestors likely underwent the same selective pressure prompted by similar geological conditions and geographic barriers. On the other hand, our results point out that the origin of *S. atlantica* is more recent in respect of the differentiation of *S. fluviatilis* and *S*. cf. *fluviatilis*, dating back to the interglacial period (approximately 390 kya) between the end of Mindel and the beginning of Riss glaciations.

## 5. Conclusions

In the present study, the hypervariable mitochondrial Control Region was shown to be highly informative in depicting the phylogeographic patterns of Italian populations of *Salariopsis fluviatilis sensu stricto* across the Ligurian Alpine ridge and the Sardinia Island-mainland dispersal patterns, with an origin and evolutionary trajectory of founders. In particular, this research contributes to put another piece in the mosaic of the molecular data of the Italian populations and helped to understand the processes of genetic differentiation involving Mediterranean island populations whose limited effective size and high degree of fragmentation make them ideal reference models for evolutionary processes.

Furthermore, the new sequences we obtained and the use of five species delimitation methods helped to obtain a simultaneous uprising of distinct taxonomic entities during the Pleistocene glaciations within the Mediterranean freshwater fish belonging to the genus *Salariopsis*. Indeed, our results support the possible occurrence of a complex of species for *S. fluviatilis*, namely *S.* cf. *fluviatilis* (as already suggested in Belaiba et al. [15]) in the Middle East regions and the Guadiana River basin taxonomic entity. In the future, the use of a combined approach with mitochondrial and nuclear markers (e.g., microsatellites, SNPs) to expand genetic variation analyses may be considered [36,37]. Indeed, previous studies on *S. fluviatilis* [9,10,20,25] evidenced that microsatellites are efficient tools in genetic monitoring to evidence genetic differences among and within populations and to highlight recent speciation processes [91].

## Figures and Tables

**Table 1:** Indices of genetic variation. The table reports the estimates of genetic variation for the mitochondrial datasets analyzed in the present study. N: sample sizes; bp: fragment size; S: number of polymorphic sites; H: number of haplotypes; h: haplotype diversity; π: nucleotide diversity.

Control Region Dataset						
	N	bp	H	S	h	π
Present study	103	304	14	16	0.554	0.00461
Sardinia	47	304	4	4	0.272	0.00046
Liguria	44	304	8	7	0.581	0.00298
Piedmont	10	304	4	4	0.644	0.00317
Lombardy	2	304	1	0	0.000	0.00000
16s Dataset						
	N	bp	H	S	h	π
Present study	103	599	8	7	0.620	0.00073
Sardinia	47	599	3	2	0.581	0.00029
Liguria	44	599	5	4	0.216	0.00041
Piedmont	10	599	2	1	0.200	0.00037
Lombardy	2	599	1	0	0.000	0.00000
Concatenated Dataset						
	N	bp	H	S	h	π
Present study	103	849	20	23	0.797	0.00210
Sardinia	47	849	7	6	0.692	0.00038
Liguria	44	849	12	11	0.644	0.00132
Piedmont	10	849	4	5	0.644	0.00136
Lombardy	2	849	1	0	0.000	0.00000

## Data Availability

Sequences obtained in the present study for the mitochondrial Control Region and 16s gene isolated in the present study were deposited in the GenBank database under the accession numbers OP675744-OP675846 and OP653611-OP653713, respectively.

## Supplementary Materials

The following supporting information can be downloaded at: https://www.mdpi.com/article/10.3390/ani12233403/s1, Figure S1. Likelihood mapping of the Control Region (A), 16s (B), and concatenated (C) dataset. The three trapezoids at the corners represent the areas supporting strictly bifurcating trees (the occurrence of a tree-like phylogenetic signal). The three rectangles on the sides represent regions where the decision between two trees is not obvious. The center of the triangle represents the region where all three unrooted trees are equally supported. Figure S2. Bayesian phylogenetic tree based on whole Control Region dataset. Values of node supports are expressed as posterior probabilities. Sequences from the present study are in red font. Figure S3. Ultrametric tree obtained by the software Beast on the whole Control Region dataset. It shows divergence time among taxa. Values of divergence time at the main node of the tree are rounded to the second decimal place. Figure S4. Median-joining network analysis performed on the whole 16s dataset. The number of mutations between sequences that are greater than *n* = 1 are reported on network branches. The number of individuals showing the same haplotype that is greater than *n* = 1 is reported inside the spots. Table S1. *Salariopsis fluviatilis* sampled specimens. The table reports data on the sampling collection and the GenBank accession numbers of the sequences obtained in the present study. Table S2. Control Region dataset. The table reports the Control Region sequences used in the present study that were taken from the GenBank database. Table S3. 16s dataset. Whole dataset including the 16s sequences taken from GenBank. Table S4. Species delimitations analyses. The table reports the results obtained from the species delimitation methods for the analysis of the whole Control Region dataset. Specimens with identical numbers within the same column belong to the same taxonomic entity. Table S5. Principal coordinates analysis. The table reports the results of the principal coordinates analysis performed on the dataset including the Control Region sequences obtained in the present study. Table S6. Principal coordinates analysis. The table reports results obtained from the principal coordinates analysis performed on the whole Control Region dataset.
