# Supplementary material for: Mitochondrial DNA of Sardinian and North-West Italian Populations Revealed a New Piece in the Mosaic of Phylogeography and Phylogeny of Salariopsis fluviatilis (Blenniidae)
Source: Animals (Basel). 2022 Dec 2;12(23):3403. doi: 10.3390/ani12233403 (PMC9736072; doi:10.3390/ani12233403)
Supplement: Supplementary file 1 [file animals-12-03403-s001.zip › Figure S2.pdf]

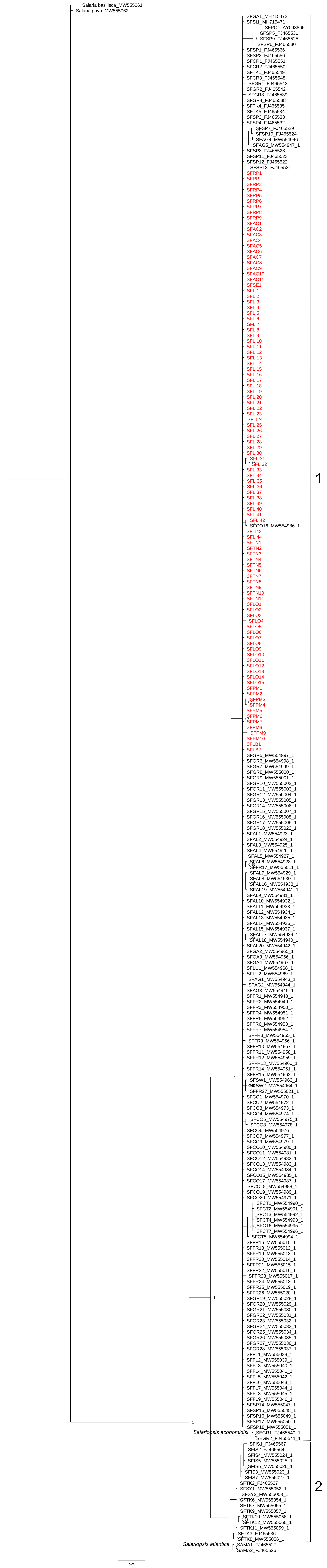

Salaria basilisca_MW555061
Salaria pavo_MW555062
SFGA1_MH715472
SFSI1_MH715471
SFPO1_AY098865
SFSP5_FJ465531
SFSP9_FJ465525
SFSP6_FJ465530
SFSP1_FJ465566
SFSP2_FJ465556
SFCR1_FJ465551
SFCR2_FJ465550
SFTK1_FJ465549
SFCR3_FJ465548
SFGR1_FJ465543
SFGR2_FJ465542
SFGR3_FJ465539
SFGR4_FJ465538
SFTK4_FJ465535
SFTK5_FJ465534
SFSP3_FJ465533
SFSP4_FJ465532
SFSP7_FJ465529
SFSP10_FJ465524
SFAG4_MW554946_1
SFAG5_MW554947_1
SFSP8_FJ465528
SFSP11_FJ465523
SFSP12_FJ465522
SFSP13_FJ465521
SFRP1
SFRP2
SFRP3
SFRP4
SFRP5
SFRP6
SFRP7
SFRP8
SFRP9
SFAC1
SFAC2
SFAC3
SFAC4
SFAC5
SFAC6
SFAC7
SFAC8
SFAC9
SFAC10
SFAC11
SFSE1
SFLI1
SFLI2
SFLI3
SFLI4
SFLI5
SFLI6
SFLI7
SFLI8
SFLI9
SFLI10
SFLI11
SFLI12
SFLI13
SFLI14
SFLI15
SFLI16
SFLI17
SFLI18
SFLI19
SFLI20
SFLI21
SFLI22
SFLI23
SFLI24
SFLI25
SFLI26
SFLI27
SFLI28
SFLI29
SFLI30
SFLI31
SFLI32
SFLI33
SFLI34
SFLI35
SFLI36
SFLI37
SFLI38
SFLI39
SFLI40
SFLI41
SFLI42
SFCO16_MW554986_1
SFLI43
SFLI44
SFTN1
SFTN2
SFTN3
SFTN4
SFTN5
SFTN6
SFTN7
SFTN8
SFTN9
SFTN10
SFTN11
SFLO1
SFLO2
SFLO3
SFLO4
SFLO5
SFLO6
SFLO7
SFLO8
SFLO9
SFLO10
SFLO11
SFLO12
SFLO13
SFLO14
SFLO15
SFPM1
SFPM2
SFPM3
SFPM4
SFPM5
SFPM6
SFPM7
SFPM8
SFPM9
SFPM10
SFLB1
SFLB2
SFGR5_MW554997_1
SFGR6_MW554998_1
SFGR7_MW554999_1
SFGR8_MW555000_1
SFGR9_MW555001_1
SFGR10_MW555002_1
SFGR11_MW555003_1
SFGR12_MW555004_1
SFGR13_MW555005_1
SFGR14_MW555006_1
SFGR15_MW555007_1
SFGR16_MW555008_1
SFGR17_MW555009_1
SFGR18_MW555022_1
SFAL1_MW554923_1
SFAL2_MW554924_1
SFAL3_MW554925_1
SFAL4_MW554926_1
SFAL5_MW554927_1
SFAL6_MW554928_1
SFFR17_MW555011_1
SFAL7_MW554929_1
SFAL8_MW554930_1
SFAL16_MW554938_1
SFAL19_MW554941_1
SFAL9_MW554931_1
SFAL10_MW554932_1
SFAL11_MW554933_1
SFAL12_MW554934_1
SFAL13_MW554935_1
SFAL14_MW554936_1
SFAL15_MW554937_1
SFAL17_MW554939_1
SFAL18_MW554940_1
SFAL20_MW554942_1
SFGA2_MW554965_1
SFGA3_MW554966_1
SFGA4_MW554967_1
SFLU1_MW554968_1
SFLU2_MW554969_1
SFAG1_MW554943_1
SFAG2_MW554944_1
SFAG3_MW554945_1
SFFR1_MW554948_1
SFFR2_MW554949_1
SFFR3_MW554950_1
SFFR4_MW554951_1
SFFR5_MW554952_1
SFFR6_MW554953_1
SFFR7_MW554954_1
SFFR8_MW554955_1
SFFR9_MW554956_1
SFFR10_MW554957_1
SFFR11_MW554958_1
SFFR12_MW554959_1
SFFR13_MW554960_1
SFFR14_MW554961_1
SFFR15_MW554962_1
SFSW1_MW554963_1
SFSW2_MW554964_1
SFFR27_MW555021_1
SFCO1_MW554970_1
SFCO2_MW554972_1
SFCO3_MW554973_1
SFCO4_MW554974_1
SFCO5_MW554975_1
SFCO8_MW554978_1
SFCO6_MW554976_1
SFCO7_MW554977_1
SFCO9_MW554979_1
SFCO10_MW554980_1
SFCO11_MW554981_1
SFCO12_MW554982_1
SFCO13_MW554983_1
SFCO14_MW554984_1
SFCO15_MW554985_1
SFCO17_MW554987_1
SFCO18_MW554988_1
SFCO19_MW554989_1
SFCO20_MW554971_1
SFCT1_MW554990_1
SFCT2_MW554991_1
SFCT3_MW554992_1
SFCT4_MW554993_1
SFCT6_MW554995_1
SFCT7_MW554996_1
SFCT5_MW554994_1
SFFR16_MW555010_1
SFFR18_MW555012_1
SFFR19_MW555013_1
SFFR20_MW555014_1
SFFR21_MW555015_1
SFFR22_MW555016_1
SFFR23_MW555017_1
SFFR24_MW555018_1
SFFR25_MW555019_1
SFFR26_MW555020_1
SFGR19_MW555028_1
SFGR20_MW555029_1
SFGR21_MW555030_1
SFGR22_MW555031_1
SFGR23_MW555032_1
SFGR24_MW555033_1
SFGR25_MW555034_1
SFGR26_MW555035_1
SFGR27_MW555036_1
SFGR28_MW555037_1
SFFL1_MW555038_1
SFFL2_MW555039_1
SFFL3_MW555040_1
SFFL4_MW555041_1
SFFL5_MW555042_1
SFFL6_MW555043_1
SFFL7_MW555044_1
SFFL8_MW555045_1
SFFL9_MW555046_1
SFSP14_MW555047_1
SFSP15_MW555048_1
SFSP16_MW555049_1
SFSP17_MW555050_1
SFSP18_MW555051_1
SEGR1_FJ465540_1
SEGR2_FJ465541_1
Salariopsis economidisi
SFIS1_FJ465567
SFIS2_FJ465564
SFIS4_MW555024_1
SFIS5_MW555025_1
SFIS6_MW555026_1
SFIS3_MW555023_1
SFIS7_MW555027_1
SFTK2_FJ465537
SFSY1_MW555052_1
SFSY2_MW555053_1
SFTK6_MW555054_1
SFTK7_MW555055_1
SFTK9_MW555057_1
SFTK10_MW555058_1
SFTK12_MW555060_1
SFTK11_MW555059_1
SFTK3_FJ465536
SFTK8_MW555056_1
Salariopsis atlantica
SAMA1_FJ465527
SAMA2_FJ465526
1
2
1
1
1
0.03
