# Supplementary material for: Mitochondrial DNA of Sardinian and North-West Italian Populations Revealed a New Piece in the Mosaic of Phylogeography and Phylogeny of Salariopsis fluviatilis (Blenniidae)
Source: Animals (Basel). 2022 Dec 2;12(23):3403. doi: 10.3390/ani12233403 (PMC9736072; doi:10.3390/ani12233403)
Supplement: Supplementary file 1 [file animals-12-03403-s001.zip › Table S1.pdf]

Table S1. *Salariopsis fluviatilis* sampled specimens. The table reports data on the sampling collection and the GenBank accession numbers of the sequences obtained in the present study.

Sample code	# Specimen collected	Administrative region	Sampling site	Sampling date	Control Region GB#	16s GB#
SFPM1 - SFPM 2	2	Piedmont	San Giovanni river	February 2019	OP675837 - OP675838	OP653704 - OP653705
SFPM3 - SFPM4	2	Piedmont	San Bernardino river	February 2019	OP675839 - OP675840	OP653706 - OP653707
SFPM5 - SFPM10	6	Piedmont	Strona di Omegna	February 2019	OP675841 - OP675846	OP653708 - OP653713
SFAC1 - SFAC11	11	Sardinia	Accu terrale creek	June 2019	OP675826 - OP675836	OP653693 - OP653703
SFRP1 - SFRP9	9	Sardinia	Riu Pale creek	June 2019	OP675816 - OP675824	OP653683 - OP653691
SFSE1	1	Sardinia	Sicaderba creek	June 2019	OP675825	OP653692
SFLI1 - SFLI36	36	Liguria	Entella river - loc. Ponte della Maddalena	July 2019	OP675770 - OP675805	OP653637 - OP653672
SFLB1 - SFLB2	2	Lombardy	Tartaro Fuga creek (Oglio river tributary)	October 2020	OP675814 - OP675815	OP653681 - OP653682
SFTN1 – SFTN11	11	Sardinia	Rio Mannu di Scano Montiferro river	June 2021	OP675744 - OP675754	OP653611 - OP653621
SFLO1 – SFLO15	15	Sardinia	Rio Mannu di Posada river	June 2021	OP675755 - OP675769	OP653622 - OP653636
SFLI37 – SFLI38	2	Liguria	Roja river - Ventimiglia	September 2021	OP675806 - OP675807	OP653673 - OP653674
SFLI39 – SFLI44	6	Liguria	Roja river – Ventimiglia – Loc. Trucco	March 2022	OP675808 - OP675813	OP653675 - OP653680
