# Supplementary material for: Mitochondrial DNA of Sardinian and North-West Italian Populations Revealed a New Piece in the Mosaic of Phylogeography and Phylogeny of Salariopsis fluviatilis (Blenniidae)
Source: Animals (Basel). 2022 Dec 2;12(23):3403. doi: 10.3390/ani12233403 (PMC9736072; doi:10.3390/ani12233403)
Supplement: Supplementary file 1 [file animals-12-03403-s001.zip › Table S2.pdf]

Table S2. Control Region dataset. The table reports the Control Region sequences used in the present study that were taken from the GenBank database.

Sample code	Sampling area	Sampling site	Species	Control Region (GB #)	Reference
S. basilisca	Greece	Chamolia	*S. basilisca*	MW555061	Wagner et al. [16]
S. pavo	Greece	Rafina river	*S. pavo*	MW555062	Wagner et al. [16]
SFAL1	Albania	Bicait river	*S. fluviatilis*	MW554923	Wagner et al. [16]
SFAL2	Albania	Bicait river	*S. fluviatilis*	MW554924	Wagner et al. [16]
SFAL3	Albania	Bicait river	*S. fluviatilis*	MW554925	Wagner et al. [16]
SFAL4	Albania	Bicait river	*S. fluviatilis*	MW554926	Wagner et al. [16]
SFAL5	Albania	Bicait river	*S. fluviatilis*	MW554927	Wagner et al. [16]
SFAL6	Albania	Bicait river	*S. fluviatilis*	MW554928	Wagner et al. [16]
SFAL7	Albania	Bicait river	*S. fluviatilis*	MW554929	Wagner et al. [16]
SFAL8	Albania	Bicait river	*S. fluviatilis*	MW554930	Wagner et al. [16]
SFAL9	Albania	Bicait river	*S. fluviatilis*	MW554931	Wagner et al. [16]
SFAL10	Albania	Bicait river	*S. fluviatilis*	MW554932	Wagner et al. [16]
SFAL11	Albania	Borshi river	*S. fluviatilis*	MW554933	Wagner et al. [16]
SFAL12	Albania	Borshi river	*S. fluviatilis*	MW554934	Wagner et al. [16]
SFAL13	Albania	Borshi river	*S. fluviatilis*	MW554935	Wagner et al. [16]
SFAL14	Albania	Borshi river	*S. fluviatilis*	MW554936	Wagner et al. [16]
SFAL15	Albania	Borshi river	*S. fluviatilis*	MW554937	Wagner et al. [16]
SFAL16	Albania	Drin Basin river	*S. fluviatilis*	MW554938	Wagner et al. [16]
SFAL17	Albania	Drin Basin river	*S. fluviatilis*	MW554939	Wagner et al. [16]
SFAL18	Albania	Drin Basin river	*S. fluviatilis*	MW554940	Wagner et al. [16]
SFAL19	Albania	Drin Basin river	*S. fluviatilis*	MW554941	Wagner et al. [16]
SFAL20	Albania	Drin Basin river	*S. fluviatilis*	MW554942	Wagner et al. [16]
SFAG1	Algeria	Oued Boughzazenne river	*S. fluviatilis*	MW554943	Wagner et al. [16]
SFAG2	Algeria	Oued Boughzazenne river	*S. fluviatilis*	MW554944	Wagner et al. [16]
SFAG3	Algeria	Oued Boughzazenne river	*S. fluviatilis*	MW554945	Wagner et al. [16]
SFAG4	Algeria	Oued Boughzazenne river	*S. fluviatilis*	MW554946	Wagner et al. [16]
SFAG5	Algeria	Oued Boughzazenne river	*S. fluviatilis*	MW554947	Wagner et al. [16]
SFFR1	France	Lake Annecy	*S. fluviatilis*	MW554948	Wagner et al. [16]
SFFR2	France	Lake Annecy	*S. fluviatilis*	MW554949	Wagner et al. [16]
SFFR3	France	Lake Annecy	*S. fluviatilis*	MW554950	Wagner et al. [16]
SFFR4	France	Lake Annecy	*S. fluviatilis*	MW554951	Wagner et al. [16]
SFFR5	France	Lake Annecy	*S. fluviatilis*	MW554952	Wagner et al. [16]
SFFR6	France	Lake Bourget	*S. fluviatilis*	MW554953	Wagner et al. [16]
SFFR7	France	Lake Bourget	*S. fluviatilis*	MW554954	Wagner et al. [16]
SFFR8	France	Lake Bourget	*S. fluviatilis*	MW554955	Wagner et al. [16]
SFFR9	France	Lake Bourget	*S. fluviatilis*	MW554956	Wagner et al. [16]
SFFR10	France	Lake Bourget	*S. fluviatilis*	MW554957	Wagner et al. [16]
SFFR11	France	Lake Aiguebelette	*S. fluviatilis*	MW554958	Wagner et al. [16]
SFFR12	France	Lake Aiguebelette	*S. fluviatilis*	MW554959	Wagner et al. [16]
SFFR13	France	Lake Aiguebelette	*S. fluviatilis*	MW554960	Wagner et al. [16]
SFFR14	France	Lake Aiguebelette	*S. fluviatilis*	MW554961	Wagner et al. [16]
SFFR15	France	Lake Aiguebelette	*S. fluviatilis*	MW554962	Wagner et al. [16]

SFFR16	France	Var river	*S. fluviatilis*	MW555010	Wagner et al. [16]
SFFR17	France	Var river	*S. fluviatilis*	MW555011	Wagner et al. [16]
SFFR18	France	Var river	*S. fluviatilis*	MW555012	Wagner et al. [16]
SFFR19	France	Var river	*S. fluviatilis*	MW555013	Wagner et al. [16]
SFFR20	France	Var river	*S. fluviatilis*	MW555014	Wagner et al. [16]
SFFR21	France	Lake Camargue	*S. fluviatilis*	MW555015	Wagner et al. [16]
SFFR22	France	Lake Camargue	*S. fluviatilis*	MW555016	Wagner et al. [16]
SFFR23	France	Tech river	*S. fluviatilis*	MW555017	Wagner et al. [16]
SFFR24	France	Tech river	*S. fluviatilis*	MW555018	Wagner et al. [16]
SFFR25	France	Tech river	*S. fluviatilis*	MW555019	Wagner et al. [16]
SFFR26	France	Tech river	*S. fluviatilis*	MW555020	Wagner et al. [16]
SFFR27	France	Rhône river	*S. fluviatilis*	MW555021	Wagner et al. [16]
SFCO1	France - Corsica	Fangu river	*S. fluviatilis*	MW554970	Wagner et al. [16]
SFCO2	France - Corsica	Fangu river	*S. fluviatilis*	MW554972	Wagner et al. [16]
SFCO3	France - Corsica	Fangu river	*S. fluviatilis*	MW554973	Wagner et al. [16]
SFCO4	France - Corsica	Fangu river	*S. fluviatilis*	MW554974	Wagner et al. [16]
SFCO5	France - Corsica	Rizzanese river	*S. fluviatilis*	MW554975	Wagner et al. [16]
SFCO6	France - Corsica	Rizzanese river	*S. fluviatilis*	MW554976	Wagner et al. [16]
SFCO7	France - Corsica	Rizzanese river	*S. fluviatilis*	MW554977	Wagner et al. [16]
SFCO8	France - Corsica	Rizzanese river	*S. fluviatilis*	MW554978	Wagner et al. [16]
SFCO9	France - Corsica	Rizzanese river	*S. fluviatilis*	MW554979	Wagner et al. [16]
SFCO10	France - Corsica	Golu river	*S. fluviatilis*	MW554980	Wagner et al. [16]
SFCO11	France - Corsica	Golu river	*S. fluviatilis*	MW554981	Wagner et al. [16]
SFCO12	France - Corsica	Golu river	*S. fluviatilis*	MW554982	Wagner et al. [16]
SFCO13	France - Corsica	Golu river	*S. fluviatilis*	MW554983	Wagner et al. [16]
SFCO14	France - Corsica	Golu river	*S. fluviatilis*	MW554984	Wagner et al. [16]
SFCO15	France - Corsica	Abatescu river	*S. fluviatilis*	MW554985	Wagner et al. [16]
SFCO16	France - Corsica	Abatescu river	*S. fluviatilis*	MW554986	Wagner et al. [16]
SFCO17	France - Corsica	Abatescu river	*S. fluviatilis*	MW554987	Wagner et al. [16]
SFCO18	France - Corsica	Abatescu river	*S. fluviatilis*	MW554988	Wagner et al. [16]
SFCO19	France - Corsica	Abatescu river	*S. fluviatilis*	MW554989	Wagner et al. [16]
SFCO20	France - Corsica	Fangu river	*S. fluviatilis*	MW554971	Wagner et al. [16]
SFSW1	Switzerland	Lake Léman (aka Geneva)	*S. fluviatilis*	MW554963	Wagner et al. [16]
SFSW2	Switzerland	Lake Léman (aka Geneva)	*S. fluviatilis*	MW554964	Wagner et al. [16]
SFGA1	Italy	Lake Garda	*S. fluviatilis*	MH715472	Belaiba et al. [15]
SFGA2	Italy	Lake Garda	*S. fluviatilis*	MW554965	Wagner et al. [16]
SFGA3	Italy	Lake Garda	*S. fluviatilis*	MW554966	Wagner et al. [16]
SFGA4	Italy	Lake Garda	*S. fluviatilis*	MW554967	Wagner et al. [16]
SFLU1	Italy	Lake Lugano	*S. fluviatilis*	MW554968	Wagner et al. [16]
SFLU2	Italy	Lake Lugano	*S. fluviatilis*	MW554969	Wagner et al. [16]
SFFL1	Italy - Sardinia	Flumendosa river	*S. fluviatilis*	MW555038	Wagner et al. [16]
SFFL2	Italy - Sardinia	Flumendosa river	*S. fluviatilis*	MW555039	Wagner et al. [16]
SFFL3	Italy - Sardinia	Flumendosa river	*S. fluviatilis*	MW555040	Wagner et al. [16]
SFFL4	Italy - Sardinia	Flumendosa river	*S. fluviatilis*	MW555041	Wagner et al. [16]
SFFL5	Italy - Sardinia	Flumendosa river	*S. fluviatilis*	MW555042	Wagner et al. [16]
SFFL6	Italy - Sardinia	Flumendosa river	*S. fluviatilis*	MW555043	Wagner et al. [16]

SFFL7	Italy - Sardinia	Flumendosa river	*S. fluviatilis*	MW555044	Wagner et al. [16]
SFFL8	Italy - Sardinia	Flumendosa river	*S. fluviatilis*	MW555045	Wagner et al. [16]
SFFL9	Italy - Sardinia	Flumendosa river	*S. fluviatilis*	MW555046	Wagner et al. [16]
SFSI1	Italy - Sicily	Frattina river	*S. fluviatilis*	MH715471	Belaiba et al. [15]
SFGR5	Greece - Euboea	Komito river	*S. fluviatilis*	MW554997	Wagner et al. [16]
SFGR6	Greece - Euboea	Komito river	*S. fluviatilis*	MW554998	Wagner et al. [16]
SFGR7	Greece - Euboea	Komito river	*S. fluviatilis*	MW554999	Wagner et al. [16]
SFGR8	Greece - Euboea	Komito river	*S. fluviatilis*	MW555000	Wagner et al. [16]
SFGR9	Greece - Euboea	Komito river	*S. fluviatilis*	MW555001	Wagner et al. [16]
SFGR10	Greece - Euboea	Komito river	*S. fluviatilis*	MW555002	Wagner et al. [16]
SFGR11	Greece - Euboea	Komito river	*S. fluviatilis*	MW555003	Wagner et al. [16]
SFGR12	Greece - Euboea	Komito river	*S. fluviatilis*	MW555004	Wagner et al. [16]
SFGR13	Greece - Euboea	Komito river	*S. fluviatilis*	MW555005	Wagner et al. [16]
SFGR14	Greece - Euboea	Komito river	*S. fluviatilis*	MW555006	Wagner et al. [16]
SFGR15	Greece - Euboea	Komito river	*S. fluviatilis*	MW555007	Wagner et al. [16]
SFGR16	Greece - Euboea	Komito river	*S. fluviatilis*	MW555008	Wagner et al. [16]
SFGR17	Greece - Euboea	Komito river	*S. fluviatilis*	MW555009	Wagner et al. [16]
SFGR18	Greece	Pinios river	*S. fluviatilis*	MW555022	Wagner et al. [16]
SFGR19	Greece	Nestos river	*S. fluviatilis*	MW555028	Wagner et al. [16]
SFGR20	Greece	Nestos river	*S. fluviatilis*	MW555029	Wagner et al. [16]
SFGR21	Greece	Nestos river	*S. fluviatilis*	MW555030	Wagner et al. [16]
SFGR22	Greece	Nestos river	*S. fluviatilis*	MW555031	Wagner et al. [16]
SFGR23	Greece	Nestos river	*S. fluviatilis*	MW555032	Wagner et al. [16]
SFGR24	Greece	Nestos river	*S. fluviatilis*	MW555033	Wagner et al. [16]
SFGR25	Greece	Nestos river	*S. fluviatilis*	MW555034	Wagner et al. [16]
SFGR26	Greece	Nestos river	*S. fluviatilis*	MW555035	Wagner et al. [16]
SFGR27	Greece	Nestos river	*S. fluviatilis*	MW555036	Wagner et al. [16]
SFGR28	Greece	Nestos river	*S. fluviatilis*	MW555037	Wagner et al. [16]
SFCT1	Greece - Crete	Lake Kournas	*S. fluviatilis*	MW554990	Wagner et al. [16]
SFCT2	Greece - Crete	Lake Kournas	*S. fluviatilis*	MW554991	Wagner et al. [16]
SFCT3	Greece - Crete	Lake Kournas	*S. fluviatilis*	MW554992	Wagner et al. [16]
SFCT4	Greece - Crete	Lake Kournas	*S. fluviatilis*	MW554993	Wagner et al. [16]
SFCT5	Greece - Crete	Lake Kournas	*S. fluviatilis*	MW554994	Wagner et al. [16]
SFCT6	Greece - Crete	Lake Kournas	*S. fluviatilis*	MW554995	Wagner et al. [16]
SFCT7	Greece - Crete	Lake Kournas	*S. fluviatilis*	MW554996	Wagner et al. [16]
SFIS3	Israel	Lake Kineret/Lower Galil Eshkol	*S. fluviatilis*	MW555023	Wagner et al. [16]
SFIS4	Israel	Lake Kineret/Lower Galil Eshkol	*S. fluviatilis*	MW555024	Wagner et al. [16]
SFIS5	Israel	Lake Kineret/Lower Galil Eshkol	*S. fluviatilis*	MW555025	Wagner et al. [16]
SFIS6	Israel	Lake Kineret/Lower Galil Eshkol	*S. fluviatilis*	MW555026	Wagner et al. [16]
SFIS7	Israel	Lake Kineret/Lower Galil Eshkol	*S. fluviatilis*	MW555027	Wagner et al. [16]
SFSP14	Spain	Segre river	*S. fluviatilis*	MW555047	Wagner et al. [16]
SFSP15	Spain	Segre river	*S. fluviatilis*	MW555048	Wagner et al. [16]
SFSP16	Spain	Segre river	*S. fluviatilis*	MW555049	Wagner et al. [16]
SFSP17	Spain	Fluvia river	*S. fluviatilis*	MW555050	Wagner et al. [16]
SFSP18	Spain	Fluvia river	*S. fluviatilis*	MW555051	Wagner et al. [16]
SFSY1	Syria	Nahr al kabir river	*S. fluviatilis*	MW555052	Wagner et al. [16]

SFSY2	Syria	Nahr al kabir river	*S. fluviatilis*	MW555053	Wagner et al. [16]
SFTK6	Turkey	Çatkıt river	*S. fluviatilis*	MW555054	Wagner et al. [16]
SFTK7	Turkey	Çatkıt river	*S. fluviatilis*	MW555055	Wagner et al. [16]
SFTK8	Turkey	Çatkıt river	*S. fluviatilis*	MW555056	Wagner et al. [16]
SFTK9	Turkey	Çatkıt river	*S. fluviatilis*	MW555057	Wagner et al. [16]
SFTK10	Turkey	Köprü river	*S. fluviatilis*	MW555058	Wagner et al. [16]
SFTK11	Turkey	Göksu river	*S. fluviatilis*	MW555059	Wagner et al. [16]
SFTK12	Turkey	Göksu river	*S. fluviatilis*	MW555060	Wagner et al. [16]
SFPO1	Portugal	Guadiana river	*S. fluviatilis*	AY098865	Almada et al. [24]
SFIS1	Israel	Lake Jordan	*S. fluviatilis*	FJ465567	Almada et al. [8]
SFSP1	Spain	Lake Banõles	*S. fluviatilis*	FJ465566	Almada et al. [8]
SFIS2	Israel	Lake Jordan	*S. fluviatilis*	FJ465564	Almada et al. [8]
SFSP2	Spain	Lake Banõles	*S. fluviatilis*	FJ465556	Almada et al. [8]
SFCR1	Croatia	Lake Bacina	*S. fluviatilis*	FJ465551	Almada et al. [8]
SFCR2	Croatia	Lake Bacina	*S. fluviatilis*	FJ465550	Almada et al. [8]
SFTK1	Turkey	Tahtal river	*S. fluviatilis*	FJ465549	Almada et al. [8]
SFCR3	Croatia	Lake Bacina	*S. fluviatilis*	FJ465548	Almada et al. [8]
SFGR1	Greece	Lake Dojranis	*S. fluviatilis*	FJ465543	Almada et al. [8]
SFGR2	Greece	Miras river	*S. fluviatilis*	FJ465542	Almada et al. [8]
SFGR3	Greece	Lake Dojranis	*S. fluviatilis*	FJ465539	Almada et al. [8]
SFGR4	Greece	Miras river	*S. fluviatilis*	FJ465538	Almada et al. [8]
SFTK2	Turkey	Çatk t river	*S. fluviatilis*	FJ465537	Almada et al. [8]
SFTK3	Turkey	Ilica river	*S. fluviatilis*	FJ465536	Almada et al. [8]
SFTK4	Turkey	Lake Iznik	*S. fluviatilis*	FJ465535	Almada et al. [8]
SFTK5	Turkey	Çak rca stream	*S. fluviatilis*	FJ465534	Almada et al. [8]
SFSP3	Spain	Matarraña river	*S. fluviatilis*	FJ465533	Almada et al. [8]
SFSP4	Spain	Noguera-Pallaresa river	*S. fluviatilis*	FJ465532	Almada et al. [8]
SFSP5	Spain	Zújar river	*S. fluviatilis*	FJ465531	Almada et al. [8]
SFSP6	Spain	Esteras river	*S. fluviatilis*	FJ465530	Almada et al. [8]
SFSP7	Spain	Verde river	*S. fluviatilis*	FJ465529	Almada et al. [8]
SFSP8	Spain	Lake Calahorra	*S. fluviatilis*	FJ465528	Almada et al. [8]
SAMA1	Morocco	Sebou river	*S. atlantica*	FJ465527	Almada et al. [8]
SAMA2	Morocco	Sebou river	*S. atlantica*	FJ465526	Almada et al. [8]
SFSP9	Spain	Zújar river	*S. fluviatilis*	FJ465525	Almada et al. [8]
SFSP10	Spain	Verde river	*S. fluviatilis*	FJ465524	Almada et al. [8]
SFSP11	Spain	Lake Calahorra	*S. fluviatilis*	FJ465523	Almada et al. [8]
SFSP12	Spain	Matarraña river	*S. fluviatilis*	FJ465522	Almada et al. [8]
SFSP13	Spain	Noguera-Pallaresa river	*S. fluviatilis*	FJ465521	Almada et al. [8]
SEGR1	Greece	Lake Trichonis	*S. economidisi*	FJ465540	Almada et al. [8]
SEGR2	Greece	Lake Trichonis	*S. economidisi*	FJ465541	Almada et al. [8]
SEGR3	Greece	Lake Trichonis	*S. economidisi*	MZ026042	Vecchioni et al. [1]
SEGR4	Greece	Lake Trichonis	*S. economidisi*	MZ026043	Vecchioni et al. [1]
SEGR5	Greece	Lake Trichonis	*S. economidisi*	MZ026044	Vecchioni et al. [1]
