# Supplementary material for: Mitochondrial DNA of Sardinian and North-West Italian Populations Revealed a New Piece in the Mosaic of Phylogeography and Phylogeny of Salariopsis fluviatilis (Blenniidae)
Source: Animals (Basel). 2022 Dec 2;12(23):3403. doi: 10.3390/ani12233403 (PMC9736072; doi:10.3390/ani12233403)
Supplement: Supplementary file 1 [file animals-12-03403-s001.zip › Table S3.pdf]

Table S3. 16s dataset. Whole dataset including the 16s sequences taken from GenBank.

**Sample code**	**Sampling area**	**Sampling site**	**Species**	**16s (GB #)**	**Reference**
S. pavo	Tunisia	Tabarka	*S. pavo*	MH724846	Belaiba et al. [15]
S. basilisca	Tunisia	La Louza	*S. basilisca*	MH724844	Belaiba et al. [15]
SFGA1	Italy	Lake Garda	*S. fluviatilis*	MH724848	Belaiba et al. [15]
SFSI1	Italy	Frattina's torrent	*S. fluviatilis*	MH724847	Belaiba et al. [15]
SFPO1	Portugal	River Guadiana	*S. fluviatilis*	AY098843	Almada et al. [24]
SFSP1	Spain	Lake Banõles	*S. fluviatilis*	FJ465748	Almada et al. [8]
SFSP2	Spain	Lake Banõles	*S. fluviatilis*	FJ465747	Almada et al. [8]
SFCR1	Croatia	-	*S. fluviatilis*	FJ465746	Almada et al. [8]
SFSP3	Spain	River Noguera-Pallaresa	*S. fluviatilis*	FJ465745	Almada et al. [8]
SFSP4	Spain	River Matarraña	*S. fluviatilis*	FJ465744	Almada et al. [8]
SFSP5	Spain	River Verde	*S. fluviatilis*	FJ465743	Almada et al. [8]
SFTK1	Turkey	River Dalaman	*S. fluviatilis*	FJ465742	Almada et al. [8]
SFSP6	Spain	River Noguera-Pallaresa	*S. fluviatilis*	FJ465741	Almada et al. [8]
SFSP7	Spain	River Matarraña	*S. fluviatilis*	FJ465740	Almada et al. [8]
SFGR1	Greece	Lake Dojranis	*S. fluviatilis*	FJ465739	Almada et al. [8]
SFGR2	Greece	Lake Dojranis	*S. fluviatilis*	FJ465738	Almada et al. [8]
SAMA1	Morocco	River Overrha	*S. atlantica*	FJ465737	Almada et al. [8]
SAMA2	Morocco	River Overrha	*S. atlantica*	FJ465736	Almada et al. [8]
SFSP8	Spain	River Zujar	*S. fluviatilis*	FJ465734	Almada et al. [8]
SFSP9	Spain	River Zujar	*S. fluviatilis*	FJ465732	Almada et al. [8]
SFTK2	Turkey	Lake Iznik	*S. fluviatilis*	FJ465731	Almada et al. [8]
SFSP10	Spain	Lake Calahorra	*S. fluviatilis*	FJ465730	Almada et al. [8]
SFGR3	Greece	River Miras	*S. fluviatilis*	FJ465729	Almada et al. [8]
SFSP11	Spain	River Esteras	*S. fluviatilis*	FJ465728	Almada et al. [8]
SFTK3	Turkey	River Ilica	*S. fluviatilis*	FJ465727	Almada et al. [8]
SFSP12	Spain	Lake Calahorra	*S. fluviatilis*	FJ465726	Almada et al. [8]
SFGR4	Greece	River Miras	*S. fluviatilis*	FJ465725	Almada et al. [8]
SFSP13	Spain	River Verde	*S. fluviatilis*	FJ465724	Almada et al. [8]
SFTK4	Turkey	River Çatk t	*S. fluviatilis*	FJ465723	Almada et al. [8]
SFIS1	Israel	-	*S. fluviatilis*	FJ465722	Almada et al. [8]
SFIS2	Israel	-	*S. fluviatilis*	FJ465718	Almada et al. [8]
SEGR1	Greece	Lake Trichonis	*S. economidisi*	FJ465735	Almada et al. [8]
SEGR2	Greece	Lake Trichonis	*S. economidisi*	FJ465733	Almada et al. [8]
