# Supplementary material for: Mitochondrial DNA of Sardinian and North-West Italian Populations Revealed a New Piece in the Mosaic of Phylogeography and Phylogeny of Salariopsis fluviatilis (Blenniidae)
Source: Animals (Basel). 2022 Dec 2;12(23):3403. doi: 10.3390/ani12233403 (PMC9736072; doi:10.3390/ani12233403)
Supplement: Supplementary file 1 [file animals-12-03403-s001.zip › Table S4.pdf]

Table S4. Species delimitations analyses. The table reports the results obtained from the species delimitation methods for the analysis of the whole Control Region dataset. Specimens with identical numbers within the same column belong to the same taxonomic entity.

Sample code	ABGD	ASAP	GMYC	PTP	NDT	Species
S. basilisca	1	1	1	4	1	*S. basilisca*
S. pavo	2	1	1	5	1	*S. pavo*
SFAL1	3	2	1	2	2	*S. fluviatilis*
SFAL2	3	2	1	2	2	*S. fluviatilis*
SFAL3	3	2	1	2	2	*S. fluviatilis*
SFAL4	3	2	1	2	2	*S. fluviatilis*
SFAL5	3	2	1	2	2	*S. fluviatilis*
SFAL6	3	2	1	2	2	*S. fluviatilis*
SFAL7	3	2	1	2	2	*S. fluviatilis*
SFAL8	3	2	1	2	2	*S. fluviatilis*
SFAL9	3	2	1	2	2	*S. fluviatilis*
SFAL10	3	2	1	2	2	*S. fluviatilis*
SFAL11	3	2	1	2	2	*S. fluviatilis*
SFAL12	3	2	1	2	2	*S. fluviatilis*
SFAL13	3	2	1	2	2	*S. fluviatilis*
SFAL14	3	2	1	2	2	*S. fluviatilis*
SFAL15	3	2	1	2	2	*S. fluviatilis*
SFAL16	3	2	1	2	2	*S. fluviatilis*
SFAL17	3	2	1	2	2	*S. fluviatilis*
SFAL18	3	2	1	2	2	*S. fluviatilis*
SFAL19	3	2	1	2	2	*S. fluviatilis*
SFAL20	3	2	1	2	2	*S. fluviatilis*
SFAG1	3	2	1	2	2	*S. fluviatilis*
SFAG2	3	2	1	2	2	*S. fluviatilis*
SFAG3	3	2	1	2	2	*S. fluviatilis*
SFAG4	3	2	1	2	2	*S. fluviatilis*
SFAG5	3	2	1	2	2	*S. fluviatilis*
SFFR1	3	2	1	2	2	*S. fluviatilis*
SFFR2	3	2	1	2	2	*S. fluviatilis*
SFFR3	3	2	1	2	2	*S. fluviatilis*
SFFR4	3	2	1	2	2	*S. fluviatilis*
SFFR5	3	2	1	2	2	*S. fluviatilis*
SFFR6	3	2	1	2	2	*S. fluviatilis*
SFFR7	3	2	1	2	2	*S. fluviatilis*
SFFR8	3	2	1	2	2	*S. fluviatilis*
SFFR9	3	2	1	2	2	*S. fluviatilis*
SFFR10	3	2	1	2	2	*S. fluviatilis*
SFFR11	3	2	1	2	2	*S. fluviatilis*
SFFR12	3	2	1	2	2	*S. fluviatilis*
SFFR13	3	2	1	2	2	*S. fluviatilis*
SFFR14	3	2	1	2	2	*S. fluviatilis*

SFFR15	3	2	1	2	2	*S. fluviatilis*
SFFR16	3	2	1	2	2	*S. fluviatilis*
SFFR17	3	2	1	2	2	*S. fluviatilis*
SFFR18	3	2	1	2	2	*S. fluviatilis*
SFFR19	3	2	1	2	2	*S. fluviatilis*
SFFR20	3	2	1	2	2	*S. fluviatilis*
SFFR21	3	2	1	2	2	*S. fluviatilis*
SFFR22	3	2	1	2	2	*S. fluviatilis*
SFFR23	3	2	1	2	2	*S. fluviatilis*
SFFR24	3	2	1	2	2	*S. fluviatilis*
SFFR25	3	2	1	2	2	*S. fluviatilis*
SFFR26	3	2	1	2	2	*S. fluviatilis*
SFFR27	3	2	1	2	2	*S. fluviatilis*
SFCO1	3	2	1	2	2	*S. fluviatilis*
SFCO2	3	2	1	2	2	*S. fluviatilis*
SFCO3	3	2	1	2	2	*S. fluviatilis*
SFCO4	3	2	1	2	2	*S. fluviatilis*
SFCO5	3	2	1	2	2	*S. fluviatilis*
SFCO6	3	2	1	2	2	*S. fluviatilis*
SFCO7	3	2	1	2	2	*S. fluviatilis*
SFCO8	3	2	1	2	2	*S. fluviatilis*
SFCO9	3	2	1	2	2	*S. fluviatilis*
SFCO10	3	2	1	2	2	*S. fluviatilis*
SFCO11	3	2	1	2	2	*S. fluviatilis*
SFCO12	3	2	1	2	2	*S. fluviatilis*
SFCO13	3	2	1	2	2	*S. fluviatilis*
SFCO14	3	2	1	2	2	*S. fluviatilis*
SFCO15	3	2	1	2	2	*S. fluviatilis*
SFCO16	3	2	1	2	2	*S. fluviatilis*
SFCO17	3	2	1	2	2	*S. fluviatilis*
SFCO18	3	2	1	2	2	*S. fluviatilis*
SFCO19	3	2	1	2	2	*S. fluviatilis*
SFCO20	3	2	1	2	2	*S. fluviatilis*
SFSW1	3	2	1	2	2	*S. fluviatilis*
SFSW2	3	2	1	2	2	*S. fluviatilis*
SFGA1	3	2	1	2	2	S. fluviatilis
SFGA2	3	2	1	2	2	*S. fluviatilis*
SFGA3	3	2	1	2	2	*S. fluviatilis*
SFGA4	3	2	1	2	2	*S. fluviatilis*
SFLU1	3	2	1	2	2	*S. fluviatilis*
SFLU2	3	2	1	2	2	*S. fluviatilis*
SFFL1	3	2	1	2	2	*S. fluviatilis*
SFFL2	3	2	1	2	2	*S. fluviatilis*
SFFL3	3	2	1	2	2	*S. fluviatilis*
SFFL4	3	2	1	2	2	*S. fluviatilis*
SFFL5	3	2	1	2	2	*S. fluviatilis*

SFFL6	3	2	1	2	2	*S. fluviatilis*
SFFL7	3	2	1	2	2	*S. fluviatilis*
SFFL8	3	2	1	2	2	*S. fluviatilis*
SFFL9	3	2	1	2	2	*S. fluviatilis*
SFSI1	3	2	1	2	2	*S. fluviatilis*
SFGR5	3	2	1	2	2	*S. fluviatilis*
SFGR6	3	2	1	2	2	*S. fluviatilis*
SFGR7	3	2	1	2	2	*S. fluviatilis*
SFGR8	3	2	1	2	2	*S. fluviatilis*
SFGR9	3	2	1	2	2	*S. fluviatilis*
SFGR10	3	2	1	2	2	*S. fluviatilis*
SFGR11	3	2	1	2	2	*S. fluviatilis*
SFGR12	3	2	1	2	2	*S. fluviatilis*
SFGR13	3	2	1	2	2	*S. fluviatilis*
SFGR14	3	2	1	2	2	*S. fluviatilis*
SFGR15	3	2	1	2	2	*S. fluviatilis*
SFGR16	3	2	1	2	2	*S. fluviatilis*
SFGR17	3	2	1	2	2	*S. fluviatilis*
SFGR18	3	2	1	2	2	*S. fluviatilis*
SFGR19	3	2	1	2	2	*S. fluviatilis*
SFGR20	3	2	1	2	2	*S. fluviatilis*
SFGR21	3	2	1	2	2	*S. fluviatilis*
SFGR22	3	2	1	2	2	*S. fluviatilis*
SFGR23	3	2	1	2	2	*S. fluviatilis*
SFGR24	3	2	1	2	2	*S. fluviatilis*
SFGR25	3	2	1	2	2	*S. fluviatilis*
SFGR26	3	2	1	2	2	*S. fluviatilis*
SFGR27	3	2	1	2	2	*S. fluviatilis*
SFGR28	3	2	1	2	2	*S. fluviatilis*
SFCT1	3	2	1	2	2	*S. fluviatilis*
SFCT2	3	2	1	2	2	*S. fluviatilis*
SFCT3	3	2	1	2	2	*S. fluviatilis*
SFCT4	3	2	1	2	2	*S. fluviatilis*
SFCT5	3	2	1	2	2	*S. fluviatilis*
SFCT6	3	2	1	2	2	*S. fluviatilis*
SFCT7	3	2	1	2	2	*S. fluviatilis*
SFIS3	5	2	1	8	4	*S. fluviatilis*
SFIS4	5	2	1	8	4	*S. fluviatilis*
SFIS5	5	2	1	8	4	*S. fluviatilis*
SFIS6	5	2	1	8	4	*S. fluviatilis*
SFIS7	5	2	1	8	4	*S. fluviatilis*
SFSP14	3	2	1	2	2	*S. fluviatilis*
SFSP15	3	2	1	2	2	*S. fluviatilis*
SFSP16	3	2	1	2	2	*S. fluviatilis*
SFSP17	3	2	1	2	2	*S. fluviatilis*
SFSP18	3	2	1	2	2	*S. fluviatilis*

SFSY1	5	2	1	7	4	*S. fluviatilis*
SFSY2	5	2	1	7	4	*S. fluviatilis*
SFTK6	5	2	1	7	4	*S. fluviatilis*
SFTK7	5	2	1	7	4	*S. fluviatilis*
SFTK8	5	2	1	6	4	*S. fluviatilis*
SFTK9	5	2	1	7	4	*S. fluviatilis*
SFTK10	5	2	1	7	4	*S. fluviatilis*
SFTK11	5	2	1	7	4	*S. fluviatilis*
SFTK12	5	2	1	7	4	*S. fluviatilis*
SFPO1	4	2	1	2	3	*S. fluviatilis*
SFIS1	5	2	1	8	4	*S. fluviatilis*
SFSP1	3	2	1	2	2	*S. fluviatilis*
SFIS2	5	2	1	8	4	*S. fluviatilis*
SFSP2	3	2	1	2	2	*S. fluviatilis*
SFCR1	3	2	1	2	2	*S. fluviatilis*
SFCR2	3	2	1	2	2	*S. fluviatilis*
SFTK1	3	2	1	2	2	*S. fluviatilis*
SFCR3	3	2	1	2	2	*S. fluviatilis*
SFGR1	3	2	1	2	2	*S. fluviatilis*
SFGR2	3	2	1	2	2	*S. fluviatilis*
SFGR3	3	2	1	2	2	*S. fluviatilis*
SFGR4	3	2	1	2	2	*S. fluviatilis*
SFTK2	5	2	1	7	4	*S. fluviatilis*
SFTK3	5	2	1	6	4	*S. fluviatilis*
SFTK4	3	2	1	2	2	*S. fluviatilis*
SFTK5	3	2	1	2	2	*S. fluviatilis*
SFSP3	3	2	1	2	2	*S. fluviatilis*
SFSP4	3	2	1	2	2	*S. fluviatilis*
SFSP5	4	2	1	2	3	*S. fluviatilis*
SFSP6	4	2	1	2	3	*S. fluviatilis*
SFSP7	3	2	1	2	2	*S. fluviatilis*
SFSP8	3	2	1	2	2	*S. fluviatilis*
SAMA1	7	2	1	1	6	*S. atlantica*
SAMA2	7	2	1	1	6	*S. atlantica*
SFSP9	4	2	1	2	3	*S. fluviatilis*
SFSP10	3	2	1	2	2	*S. fluviatilis*
SFSP11	3	2	1	2	2	*S. fluviatilis*
SFSP12	3	2	1	2	2	*S. fluviatilis*
SFSP13	3	2	1	2	2	*S. fluviatilis*
SEGR1	6	2	1	3	5	*S. economidisi*
SEGR2	6	2	1	3	5	*S. economidisi*
SEGR3	6	2	1	3	5	*S. economidisi*
SEGR4	6	2	1	3	5	*S. economidisi*
SEGR5	6	2	1	3	5	*S. economidisi*
SFAC1	3	2	1	2	2	*S. fluviatilis*
SFAC2	3	2	1	2	2	*S. fluviatilis*

SFAC3	3	2	1	2	2	*S. fluviatilis*
SFAC4	3	2	1	2	2	*S. fluviatilis*
SFAC5	3	2	1	2	2	*S. fluviatilis*
SFAC6	3	2	1	2	2	*S. fluviatilis*
SFAC7	3	2	1	2	2	*S. fluviatilis*
SFAC8	3	2	1	2	2	*S. fluviatilis*
SFAC9	3	2	1	2	2	*S. fluviatilis*
SFAC10	3	2	1	2	2	*S. fluviatilis*
SFAC 11	3	2	1	2	2	*S. fluviatilis*
SFRP1	3	2	1	2	2	*S. fluviatilis*
SFRP2	3	2	1	2	2	*S. fluviatilis*
SFRP3	3	2	1	2	2	*S. fluviatilis*
SFRP4	3	2	1	2	2	*S. fluviatilis*
SFRP5	3	2	1	2	2	*S. fluviatilis*
SFRP6	3	2	1	2	2	*S. fluviatilis*
SFRP7	3	2	1	2	2	*S. fluviatilis*
SFRP8	3	2	1	2	2	*S. fluviatilis*
SFRP9	3	2	1	2	2	*S. fluviatilis*
SFSE1	3	2	1	2	2	*S. fluviatilis*
SFTN1	3	2	1	2	2	*S. fluviatilis*
SFTN2	3	2	1	2	2	*S. fluviatilis*
SFTN3	3	2	1	2	2	*S. fluviatilis*
SFTN4	3	2	1	2	2	*S. fluviatilis*
SFTN5	3	2	1	2	2	*S. fluviatilis*
SFTN6	3	2	1	2	2	*S. fluviatilis*
SFTN7	3	2	1	2	2	*S. fluviatilis*
SFTN8	3	2	1	2	2	*S. fluviatilis*
SFTN9	3	2	1	2	2	*S. fluviatilis*
SFTN10	3	2	1	2	2	*S. fluviatilis*
SFTN11	3	2	1	2	2	*S. fluviatilis*
SFLO1	3	2	1	2	2	*S. fluviatilis*
SFLO2	3	2	1	2	2	*S. fluviatilis*
SFLO3	3	2	1	2	2	*S. fluviatilis*
SFLO4	3	2	1	2	2	*S. fluviatilis*
SFLO5	3	2	1	2	2	*S. fluviatilis*
SFLO6	3	2	1	2	2	*S. fluviatilis*
SFLO7	3	2	1	2	2	*S. fluviatilis*
SFLO8	3	2	1	2	2	*S. fluviatilis*
SFLO9	3	2	1	2	2	*S. fluviatilis*
SFLO10	3	2	1	2	2	*S. fluviatilis*
SFLO11	3	2	1	2	2	*S. fluviatilis*
SFLO12	3	2	1	2	2	*S. fluviatilis*
SFLO13	3	2	1	2	2	*S. fluviatilis*
SFLO14	3	2	1	2	2	*S. fluviatilis*
SFLO15	3	2	1	2	2	*S. fluviatilis*
SFLI1	3	2	1	2	2	*S. fluviatilis*

SFLI2	3	2	1	2	2	*S. fluviatilis*
SFLI3	3	2	1	2	2	*S. fluviatilis*
SFLI4	3	2	1	2	2	*S. fluviatilis*
SFLI5	3	2	1	2	2	*S. fluviatilis*
SFLI6	3	2	1	2	2	*S. fluviatilis*
SFLI7	3	2	1	2	2	*S. fluviatilis*
SFLI8	3	2	1	2	2	*S. fluviatilis*
SFLI9	3	2	1	2	2	*S. fluviatilis*
SFLI10	3	2	1	2	2	*S. fluviatilis*
SFLI11	3	2	1	2	2	*S. fluviatilis*
SFLI12	3	2	1	2	2	*S. fluviatilis*
SFLI13	3	2	1	2	2	*S. fluviatilis*
SFLI14	3	2	1	2	2	*S. fluviatilis*
SFLI15	3	2	1	2	2	*S. fluviatilis*
SFLI16	3	2	1	2	2	*S. fluviatilis*
SFLI17	3	2	1	2	2	*S. fluviatilis*
SFLI18	3	2	1	2	2	*S. fluviatilis*
SFLI19	3	2	1	2	2	*S. fluviatilis*
SFLI20	3	2	1	2	2	*S. fluviatilis*
SFLI21	3	2	1	2	2	*S. fluviatilis*
SFLI22	3	2	1	2	2	*S. fluviatilis*
SFLI23	3	2	1	2	2	*S. fluviatilis*
SFLI24	3	2	1	2	2	*S. fluviatilis*
SFLI25	3	2	1	2	2	*S. fluviatilis*
SFLI26	3	2	1	2	2	*S. fluviatilis*
SFLI27	3	2	1	2	2	*S. fluviatilis*
SFLI28	3	2	1	2	2	*S. fluviatilis*
SFLI29	3	2	1	2	2	*S. fluviatilis*
SFLI30	3	2	1	2	2	*S. fluviatilis*
SFLI31	3	2	1	2	2	*S. fluviatilis*
SFLI32	3	2	1	2	2	*S. fluviatilis*
SFLI33	3	2	1	2	2	*S. fluviatilis*
SFLI34	3	2	1	2	2	*S. fluviatilis*
SFLI35	3	2	1	2	2	*S. fluviatilis*
SFLI36	3	2	1	2	2	*S. fluviatilis*
SFLI37	3	2	1	2	2	*S. fluviatilis*
SFLI38	3	2	1	2	2	*S. fluviatilis*
SFLI39	3	2	1	2	2	*S. fluviatilis*
SFLI40	3	2	1	2	2	*S. fluviatilis*
SFLI41	3	2	1	2	2	*S. fluviatilis*
SFLI42	3	2	1	2	2	*S. fluviatilis*
SFLI43	3	2	1	2	2	*S. fluviatilis*
SFLI44	3	2	1	2	2	*S. fluviatilis*
SFPM1	3	2	1	2	2	*S. fluviatilis*
SFPM2	3	2	1	2	2	*S. fluviatilis*
SFPM3	3	2	1	2	2	*S. fluviatilis*

SFPM4	3	2	1	2	2	*S. fluviatilis*
SFPM5	3	2	1	2	2	*S. fluviatilis*
SFPM6	3	2	1	2	2	*S. fluviatilis*
SFPM7	3	2	1	2	2	*S. fluviatilis*
SFPM8	3	2	1	2	2	*S. fluviatilis*
SFPM9	3	2	1	2	2	*S. fluviatilis*
SFPM10	3	2	1	2	2	*S. fluviatilis*
SFLB1	3	2	1	2	2	*S. fluviatilis*
SFLB2	3	2	1	2	2	*S. fluviatilis*
