# Supplementary material for: Mitochondrial DNA of Sardinian and North-West Italian Populations Revealed a New Piece in the Mosaic of Phylogeography and Phylogeny of Salariopsis fluviatilis (Blenniidae)
Source: Animals (Basel). 2022 Dec 2;12(23):3403. doi: 10.3390/ani12233403 (PMC9736072; doi:10.3390/ani12233403)
Supplement: Supplementary file 1 [file animals-12-03403-s001.zip › Table S5.pdf]

Table S5. Principal coordinates analysis. The table reports the results of the principal coordinates analysis performed on the dataset including the Control Region sequences obtained in the present study.

**Group 1**	
**Sample code**	**Sampling area**
SFPM1	Piedmont
SFPM2	Piedmont
SFPM3	Piedmont
SFPM4	Piedmont
SFPM5	Piedmont
SFPM6	Piedmont
SFPM7	Piedmont
SFPM9	Piedmont
SFPM10	Piedmont
SFLB1	Lombardy
SFLB2	Lombardy
**Group 2**	
**Sample code**	**Sampling area**
SFPM8	Piedmont
**Group 3**	
**Sample code**	**Sampling area**
SFLO4	Sardinia
**Group 4**	
**Sample code**	**Sampling area**
SFLI1	Liguria
SFLI9	Liguria
SFLI22	Liguria
SFLI23	Liguria
**Group 5**	
**Sample code**	**Sampling area**
SFLI2	Liguria
SFLI3	Liguria
SFLI4	Liguria
SFLI5	Liguria
SFLI6	Liguria
SFLI7	Liguria
SFLI8	Liguria
SFLI10	Liguria
SFLI11	Liguria
SFLI12	Liguria
SFLI13	Liguria
SFLI14	Liguria
SFLI15	Liguria
SFLI16	Liguria
SFLI17	Liguria
SFLI18	Liguria
SFLI19	Liguria
SFLI20	Liguria
SFLI21	Liguria
SFLI24	Liguria
SFLI25	Liguria
SFLI26	Liguria
SFLI27	Liguria
SFLI28	Liguria
SFLI29	Liguria
SFLI30	Liguria

SFLI31	Liguria
SFLI32	Liguria
SFLI33	Liguria
SFLI34	Liguria
SFLI35	Liguria
SFLI36	Liguria
SFLI38	Liguria
SFLI39	Liguria
SFLI43	Liguria
SFRP1	Sardinia
SFRP2	Sardinia
SFRP3	Sardinia
SFRP4	Sardinia
SFRP5	Sardinia
SFRP6	Sardinia
SFRP7	Sardinia
SFRP8	Sardinia
SFRP9	Sardinia
SFAC1	Sardinia
SFAC2	Sardinia
SFAC3	Sardinia
SFAC4	Sardinia
SFAC5	Sardinia
SFAC6	Sardinia
SFAC7	Sardinia
SFAC8	Sardinia
SFAC9	Sardinia
SFAC10	Sardinia
SFAC11	Sardinia
SFSE1	Sardinia
SFTN1	Sardinia
SFTN2	Sardinia
SFTN3	Sardinia
SFTN5	Sardinia
SFTN6	Sardinia
SFTN7	Sardinia
SFTN9	Sardinia
SFTN10	Sardinia
SFTN11	Sardinia
SFLO1	Sardinia
SFLO2	Sardinia
SFLO5	Sardinia
SFLO6	Sardinia
SFLO7	Sardinia
SFLO8	Sardinia
SFLO9	Sardinia
SFLO10	Sardinia
SFLO11	Sardinia
SFLO12	Sardinia
SFLO13	Sardinia
SFLO14	Sardinia
SFLO15	Sardinia
**Group 6**	
**Sample code**	**Sampling area**
SFLI37	Liguria
SFLI40	Liguria

SFLI41	Liguria
SFLI42	Liguria
SFLI44	Liguria
SFTN4	Sardinia
SFTN8	Sardinia
SFLO3	Sardinia
