# Supplementary material for: Mitochondrial DNA of Sardinian and North-West Italian Populations Revealed a New Piece in the Mosaic of Phylogeography and Phylogeny of Salariopsis fluviatilis (Blenniidae)
Source: Animals (Basel). 2022 Dec 2;12(23):3403. doi: 10.3390/ani12233403 (PMC9736072; doi:10.3390/ani12233403)
Supplement: Supplementary file 1 [file animals-12-03403-s001.zip › Table S6.pdf]

Table S6. Principal coordinates analysis. The table reports results obtained from the principal coordinates analysis performed on the whole Control Region dataset.

**Group 1**	
**Sample code**	**Country**
SFPM1	Italy - Piedmont
SFPM2	Italy - Piedmont
SFPM3	Italy - Piedmont
SFPM4	Italy - Piedmont
SFPM5	Italy - Piedmont
SFPM6	Italy - Piedmont
SFPM7	Italy - Piedmont
SFPM8	Italy - Piedmont
SFPM9	Italy - Piedmont
SFPM10	Italy - Piedmont
SFLB1	Italy - Lombardy
SFLB2	Italy - Lombardy
SFAL5	Albania
SFGA3	Italy – Lake Garda
SFGR1	Greece
SFGR2	Greece
SFGR3	Greece
SFGR4	Greece
SFTK4	Turkey
SFTK5	Turkey
SFTK1	Turkey
SFGR5	Greece
SFGR6	Greece
SFGR7	Greece
SFGR8	Greece
SFGR9	Greece
SFGR10	Greece
SFGR11	Greece
SFGR12	Greece
SFGR13	Greece
SFGR14	Greece
SFGR15	Greece
SFGR16	Greece
SFGR17	Greece
SFGR18	Greece
SFGR19	Greece
SFGR20	Greece
SFGR21	Greece
SFGR22	Greece
SFGR23	Greece
SFGR24	Greece
SFGR25	Greece
SFGR26	Greece
SFGR27	Greece
SFGR28	Greece
SFAL1	Albania
SFAL2	Albania
SFAL3	Albania
SFAL4	Albania
SFAL6	Albania
SFAL7	Albania

SFAL8	Albania
SFAL9	Albania
SFAL10	Albania
SFAL11	Albania
SFAL12	Albania
SFAL13	Albania
SFAL14	Albania
SFAL15	Albania
SFAL16	Albania
SFAL17	Albania
SFAL18	Albania
SFAL19	Albania
SFAL20	Albania
SFCR2	Croatia
SFCR3	Croatia
SFCR1	Croatia
SFGA1	Italy – Lake Garda
SFGA2	Italy – Lake Garda
SFGA4	Italy – Lake Garda
SFSI1	Italy - Sicily
SFLU1	Italy – Lake Lugano
SFLU2	Italy – Lake Lugano
**Group 2**	
**Sample code**	**Country**
SFSP1	Spain
SFSP2	Spain
SFSP3	Spain
SFSP4	Spain
SFSP7	Spain
SFSP8	Spain
SFSP10	Spain
SFSP11	Spain
SFSP12	Spain
SFSP13	Spain
SFRP1	Italy - Sardinia
SFRP2	Italy - Sardinia
SFRP3	Italy - Sardinia
SFRP4	Italy - Sardinia
SFRP5	Italy - Sardinia
SFRP6	Italy - Sardinia
SFRP7	Italy - Sardinia
SFRP8	Italy - Sardinia
SFRP9	Italy - Sardinia
SFAC1	Italy - Sardinia
SFAC2	Italy - Sardinia
SFAC3	Italy - Sardinia
SFAC4	Italy - Sardinia
SFAC5	Italy - Sardinia
SFAC6	Italy - Sardinia
SFAC7	Italy - Sardinia
SFAC8	Italy - Sardinia
SFAC9	Italy - Sardinia
SFAC10	Italy - Sardinia
SFAC11	Italy - Sardinia
SFSE1	Italy - Sardinia
SFLI1	Italy - Liguria

SFLI2	Italy - Liguria
SFLI3	Italy - Liguria
SFLI4	Italy - Liguria
SFLI5	Italy - Liguria
SFLI6	Italy - Liguria
SFLI7	Italy - Liguria
SFLI8	Italy - Liguria
SFLI9	Italy - Liguria
SFLI10	Italy - Liguria
SFLI11	Italy - Liguria
SFLI12	Italy - Liguria
SFLI13	Italy - Liguria
SFLI14	Italy - Liguria
SFLI15	Italy - Liguria
SFLI16	Italy - Liguria
SFLI17	Italy - Liguria
SFLI18	Italy - Liguria
SFLI19	Italy - Liguria
SFLI20	Italy - Liguria
SFLI21	Italy - Liguria
SFLI22	Italy - Liguria
SFLI23	Italy - Liguria
SFLI24	Italy - Liguria
SFLI25	Italy - Liguria
SFLI26	Italy - Liguria
SFLI27	Italy - Liguria
SFLI28	Italy - Liguria
SFLI29	Italy - Liguria
SFLI30	Italy - Liguria
SFLI31	Italy - Liguria
SFLI32	Italy - Liguria
SFLI33	Italy - Liguria
SFLI34	Italy - Liguria
SFLI35	Italy - Liguria
SFLI36	Italy - Liguria
SFLI37	Italy - Liguria
SFLI38	Italy - Liguria
SFLI39	Italy - Liguria
SFLI40	Italy - Liguria
SFLI41	Italy - Liguria
SFLI42	Italy - Liguria
SFLI43	Italy - Liguria
SFLI44	Italy - Liguria
SFTN1	Italy - Sardinia
SFTN2	Italy - Sardinia
SFTN3	Italy - Sardinia
SFTN4	Italy - Sardinia
SFTN5	Italy - Sardinia
SFTN6	Italy - Sardinia
SFTN7	Italy - Sardinia
SFTN8	Italy - Sardinia
SFTN9	Italy - Sardinia
SFTN10	Italy - Sardinia
SFTN11	Italy - Sardinia
SFLO1	Italy - Sardinia
SFLO2	Italy - Sardinia

SFLO3	Italy - Sardinia
SFLO4	Italy - Sardinia
SFLO5	Italy - Sardinia
SFLO6	Italy - Sardinia
SFLO7	Italy - Sardinia
SFLO8	Italy - Sardinia
SFLO9	Italy - Sardinia
SFLO10	Italy - Sardinia
SFLO11	Italy - Sardinia
SFLO12	Italy - Sardinia
SFLO13	Italy - Sardinia
SFLO14	Italy - Sardinia
SFLO15	Italy - Sardinia
SFAG1	Algeria
SFAG2	Algeria
SFAG3	Algeria
SFAG4	Algeria
SFAG5	Algeria
SFFR1	France
SFFR2	France
SFFR3	France
SFFR4	France
SFFR5	France
SFFR6	France
SFFR7	France
SFFR8	France
SFFR9	France
SFFR10	France
SFFR11	France
SFFR12	France
SFFR13	France
SFFR14	France
SFFR15	France
SFSW1	Switzerland
SFSW2	Switzerland
SFCO1	France - Corsica
SFCO2	France - Corsica
SFCO3	France - Corsica
SFCO4	France - Corsica
SFCO5	France - Corsica
SFCO6	France - Corsica
SFCO7	France - Corsica
SFCO8	France - Corsica
SFCO9	France - Corsica
SFCO10	France - Corsica
SFCO11	France - Corsica
SFCO12	France - Corsica
SFCO13	France - Corsica
SFCO14	France - Corsica
SFCO15	France - Corsica
SFCO16	France - Corsica
SFCO17	France - Corsica
SFCO18	France - Corsica
SFCO19	France - Corsica
SFCO20	France - Corsica
SFCT1	Greece - Crete

SFCT2	Greece - Crete
SFCT3	Greece - Crete
SFCT4	Greece - Crete
SFCT5	Greece - Crete
SFCT6	Greece - Crete
SFCT7	Greece - Crete
SFFR16	France
SFFR17	France
SFFR18	France
SFFR19	France
SFFR20	France
SFFR21	France
SFFR22	France
SFFR23	France
SFFR24	France
SFFR25	France
SFFR26	France
SFFR27	France
SFFL1	Italy - Sardinia
SFFL2	Italy - Sardinia
SFFL3	Italy - Sardinia
SFFL4	Italy - Sardinia
SFFL5	Italy - Sardinia
SFFL6	Italy - Sardinia
SFFL7	Italy - Sardinia
SFFL8	Italy - Sardinia
SFFL9	Italy - Sardinia
SFSP14	Spain
SFSP15	Spain
SFSP16	Spain
SFSP17	Spain
SFSP18	Spain
**Group 3**	
**Sample code**	**Country**
SFSP5	Spain
SFSP6	Spain
SFSP9	Spain
SFPO1	Portugal
**Group 4**	
**Sample code**	**Country**
SFTK7	Turkey
SFTK12	Turkey
SFTK10	Turkey
SFTK6	Turkey
SFTK8	Turkey
SFTK9	Turkey
SFSY1	Syria
SFSY2	Syria
SFTK11	Turkey
SFIS3	Israel
SFIS4	Israel
SFIS5	Israel
SFIS6	Israel
SFIS7	Israel
SFTK2	Turkey
SFTK3	Turkey

SFIS1	Israel
SFIS2	Israel
