# Supplementary figures and images for: Mitochondrial DNA of Sardinian and North-West Italian Populations Revealed a New Piece in the Mosaic of Phylogeography and Phylogeny of Salariopsis fluviatilis (Blenniidae)
Source: Animals (Basel). 2022 Dec 2;12(23):3403. doi: 10.3390/ani12233403 (PMC9736072; doi:10.3390/ani12233403)

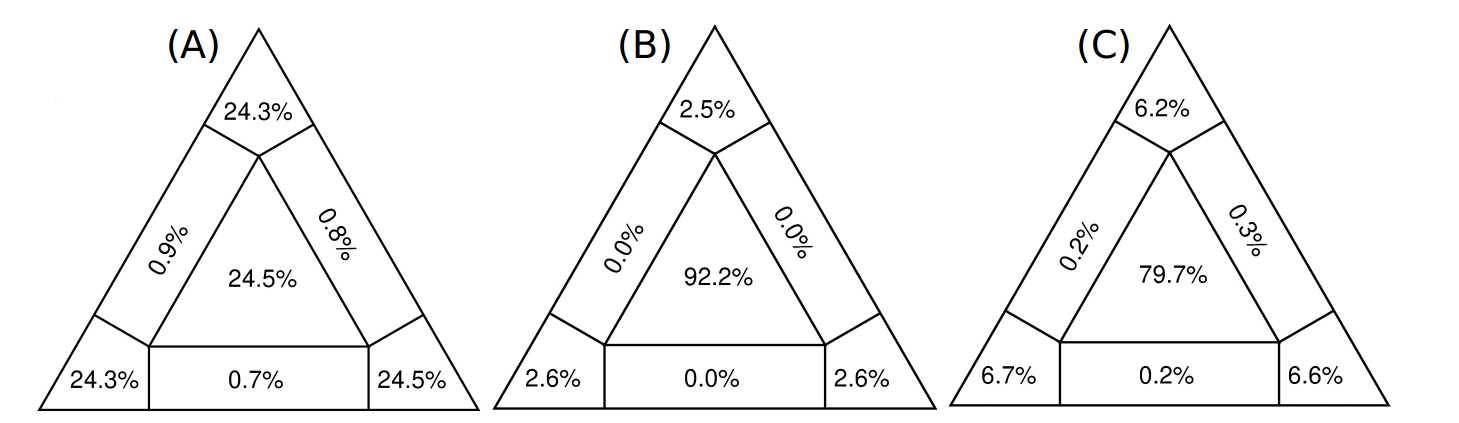

Supplement: Supplementary file 1 [file animals-12-03403-s001.zip › Figure S1.jpg]

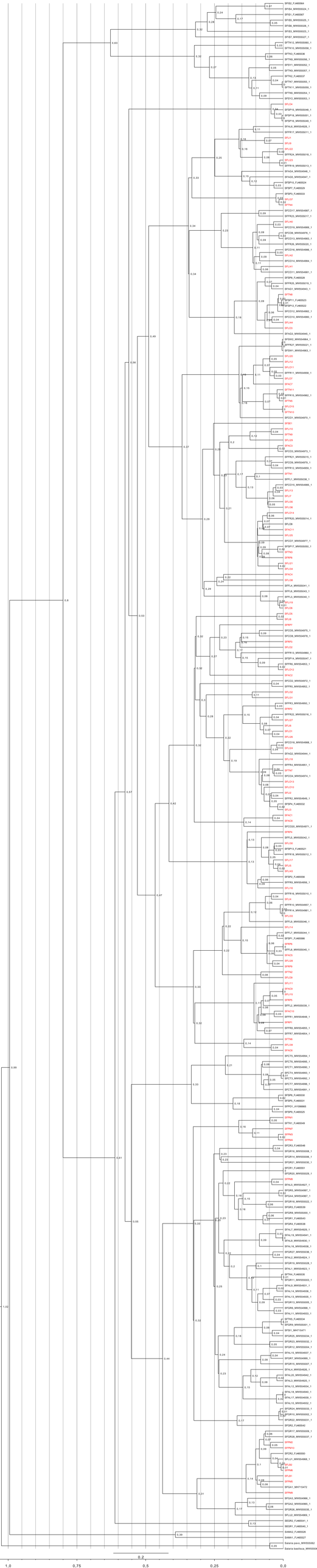

Supplement: Supplementary file 1 [file animals-12-03403-s001.zip › Figure S3.pdf]

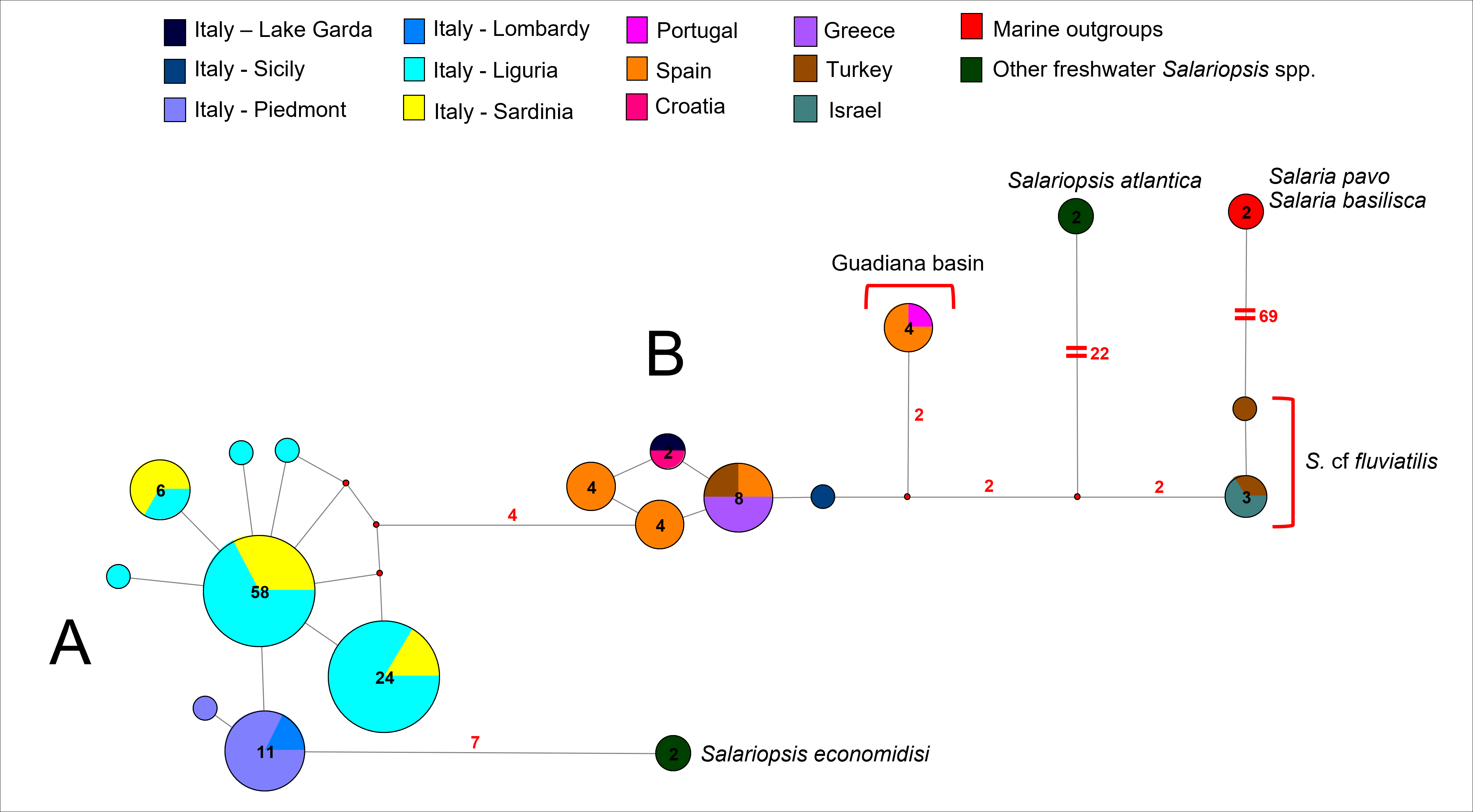

Supplement: Supplementary file 1 [file animals-12-03403-s001.zip › Figure S4.tif]
